# Acute Capillary Plasma Biomarker, Neuromuscular, and Perceptual Responses to Standardised Soccer Match Play in Elite Players: A Descriptive Study of Asynchronous Multi-Domain Recovery

**DOI:** 10.3390/metabo16060370

**Published:** 2026-05-29

**Authors:** Lun Du, Jie Xiao, Chunpeng Li, Shuning Liu, Yaji Jiang, Yue Dou, Haotian Zhao, Wen Zhong, Kai Zhao, Chang Liu

**Affiliations:** 1 Henan University of Chinese Medicine, Zhengzhou 450046, China; du378875461@gmail.com; 2 Russian State Sports University, Moscow 105122, Russia; 3 School of Sports Science, Beijing Sport University, Beijing 100084, China; 4 Graduate School of Trainers, Belarusian State University of Physical Culture, 220020 Minsk, Belarus; 5 China Football College, Beijing Sport University, Beijing 100084, China; 6 Department of Physical Education, Jiangnan University, Wuxi 214122, China; 7 China Volleyball Academy, Beijing Sport University, Beijing 100084, China

**Keywords:** soccer, elite players, capillary blood, creatine kinase, interleukin-6, cortisol, countermovement jump, sprint, fatigue

## Abstract

Background: Soccer match play induces substantial mechanical, metabolic, inflammatory, and neuromuscular stress, yet post-match monitoring in applied settings often relies on isolated markers, venous sampling, or limited time points. This observational repeated-measures study aimed to describe whether capillary-derived biomarkers, neuromuscular performance, and perceptual measures showed asynchronous recovery during the first 48 h after a standardised soccer match in elite players. Methods: Twenty-two elite male outfield soccer players completed a standardised 90 min match. Capillary blood biomarkers, countermovement jump (CMJ), 20 m sprint performance, maximal voluntary contraction (MVC), and delayed onset muscle soreness (DOMS) were assessed before the match, immediately post-match, and at 24 and 48 h post-match. Time effects were analysed using repeated-measures mixed-effects models, and associations between biochemical and functional responses were examined descriptively. Results: Match play induced clear but domain-specific disturbances. IL-6 and cortisol rose rapidly immediately post-match, whereas hsCRP, CK, LDH, myoglobin, and DOMS showed delayed peaks during early recovery. CK, LDH, myoglobin, and soreness remained above baseline at 48 h. CMJ and sprint performance were impaired after the match but largely recovered by 48 h, whereas MVC showed its greatest decrement at 24 h. Exploratory associations indicated that larger muscle damage responses tended to co-occur with greater strength and jump decrements and higher soreness, but these analyses were not causal. Conclusions: Recovery after a standardised elite soccer match was multidimensional and non-synchronous across physiological, neuromuscular, and perceptual domains. A capillary-based, multi-domain assessment strategy may provide a feasible descriptive perspective for field-based observation of post-match fatigue.

## 1. Introduction

Soccer match play involves repeated high-intensity efforts, accelerations, decelerations and changes of direction performed over a prolonged period. This intermittent demand profile requires players to repeatedly perform high-intensity actions while tolerating substantial cumulative fatigue across the match [1]. Such demands place considerable mechanical and metabolic stress on players and can lead to acute and short-lasting fatigue that influences subsequent performance and injury risk [2,3,4]. With increasingly congested competition schedules at the elite level, there is a continued need for practical and reliable indicators that help describe how players respond to match load and how quickly they recover [5,6].

A variety of biochemical, neuromuscular, and perceptual measures have been used to monitor players’ physiological responses following matches. Biochemical markers reflecting muscle damage—such as creatine kinase (CK), lactate dehydrogenase (LDH), and myoglobin—typically increase after intense exercise, indicating the extent of muscle strain [2,3,7]. Inflammatory and immune markers, including interleukin-6 (IL-6), tumour necrosis factor-α(TNF-α), high-sensitivity C-reactive protein (hs-CRP), and leukocyte counts, provide insight into the temporal dynamics of acute inflammation and recovery processes [2,3,7,8]. Endocrine and metabolic markers, such as cortisol, testosterone, the testosterone-to-cortisol ratio (T/C), lactate, urea, creatinine, and irisin, reflect stress responses and anabolic–catabolic balance, which can influence both performance and recovery [2,3,9,10].

In addition to biochemical measures, functional tests—including countermovement jump (CMJ) height, sprint performance, and isometric strength—together with subjective perceptual assessments, such as delayed onset muscle soreness (DOMS), offer complementary information on neuromuscular function and perceptual fatigue [2,3]. Meta-analyses and synthesis studies consistently report pronounced increases in CK and myoglobin, acute elevations in IL-6 followed by delayed rises in CRP, short-term disruptions in endocrine markers, and small-to-moderate declines in CMJ and sprint performance, accompanied by notable perceptual fatigue [2,3,11].

Despite this substantial body of research, an important applied gap remains. Many studies focus on a single physiological domain, rely on venous sampling that is less practical for repeated field use, or include only one or two post-match time points [2,3,7,8]. Consequently, practitioners may receive fragmented information that does not clearly show whether biochemical stress, neuromuscular performance, and perceptual recovery evolve in parallel or on different timelines. This issue is especially relevant in elite soccer, where short recovery windows require rapid but cautious interpretation of player status.

The present study was therefore designed as an applied descriptive and hypothesis-generating investigation rather than as the development or validation of a monitoring framework or model. Its intended contribution is to integrate repeated capillary blood sampling with neuromuscular and perceptual testing within the same elite cohort, thereby illustrating how different recovery domains may converge or dissociate across the first 48 h after match play [9,10,11]. In this context, capillary sampling was used to improve field feasibility and ecological relevance, not to demonstrate superiority over venous sampling or to establish diagnostic thresholds [9,11,12].

The primary research question was whether capillary-derived biomarkers reflecting muscle damage, inflammatory–immune load, and endocrine–metabolic stress would recover on the same timeline as neuromuscular performance and perceptual soreness following a standardised 90 min match in elite male soccer players. The secondary aim was to explore, descriptively, whether the magnitude of biochemical perturbation tended to co-vary with decrements in performance and soreness. The 48 h monitoring window was selected because it captures the period in which acute inflammatory responses emerge immediately after competition and muscle damage markers commonly peak during early recovery [2,9]. We hypothesised that (i) inflammatory and endocrine markers would show rapid post-match disturbances and earlier normalisation; (ii) muscle damage indices, maximal strength loss, and soreness would show delayed maximal impairment around 24 h; and (iii) responses across domains would be only partially aligned rather than fully synchronous. A field-feasible design based on repeated capillary sampling and simple performance testing may therefore provide a practical descriptive illustration of how different physiological, neuromuscular, and perceptual recovery domains evolve over time in an applied elite soccer context.

## 2. Methods

### 2.1. Study Design and Participants

This observational repeated-measures study was conducted in a single elite men’s soccer squad competing in the Chinese Football Association China League. Twenty-two outfield players volunteered and completed all testing sessions. Inclusion criteria were: (i) full participation in team training and matches during the preceding month; (ii) absence of current musculoskeletal injury or illness; and (iii) no use of anti-inflammatory medication or nutritional supplements known to influence muscle damage or inflammatory markers within the 2 weeks before data collection. No formal a priori power calculation was performed. The sample size was determined by the number of eligible players available within the squad and by the practical constraints of repeated field-based testing in an elite team environment. Accordingly, the statistical analyses should be interpreted as descriptive and exploratory rather than confirmatory.

All participants belonged to the same squad and were exposed to similar team-level training routines, tactical instructions, and recovery practices, which helped reduce some contextual variability. A within-subject longitudinal design with standardised match exposure was used because it is suitable for describing time-dependent post-match changes within an applied elite setting. However, no non-match control condition or alternative recovery condition was included. Therefore, this study was designed to characterise within-player recovery kinetics after a standardised match exposure, not to isolate the causal effect of match play from all other potential influences or to support broad population-level inference.

This study was approved by the local ethics committee, and written informed consent was obtained from all participants. All procedures were conducted in accordance with the Declaration of Helsinki [13].

### 2.2. Match Protocol and Overall Procedures

Players completed a standardised 11 vs. 11 training match lasting 90 min (2 × 45 min) on a full-size natural grass pitch scheduled at the team’s habitual training time. Warm-up procedures, tactical instructions, and substitution rules followed the club’s usual practice to maximise ecological validity within a controlled applied setting. Ambient conditions and pitch context were noted in field records, but temperature, humidity, wind, and surface conditions were not continuously monitored or incorporated as covariates in the statistical models. This limited environmental reporting should be considered when interpreting biomarker and performance responses.

Objective external and internal match load metrics were not collected. Specifically, GPS-derived total distance, high-speed running distance, sprint count, accelerations/decelerations, player load, and match heart rate responses were unavailable. Therefore, the physiological responses reported here should be interpreted in relation to a standardised match exposure rather than to quantified individual workload, and inter-individual variability cannot be directly attributed to specific external or internal load demands.

**Figure 1:**
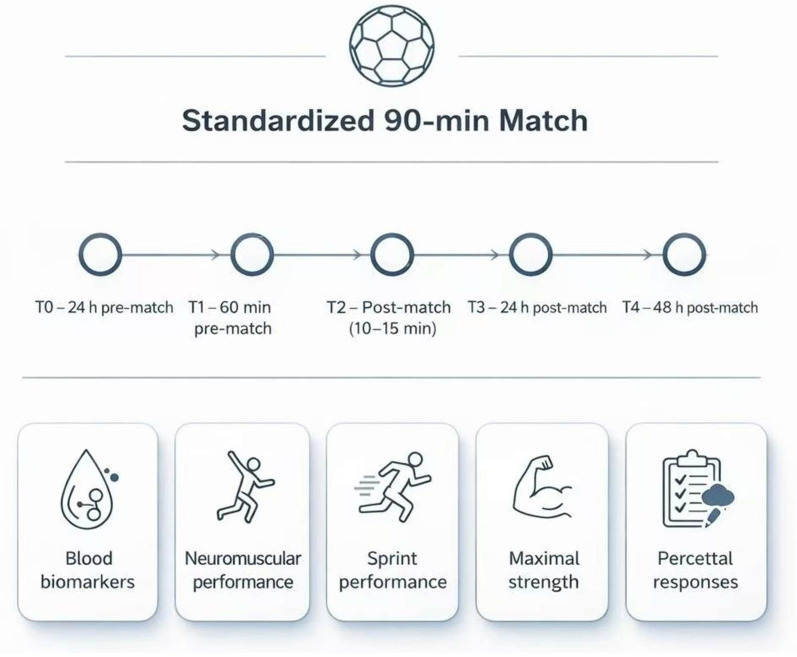
Testing workflow and measurement timeline following a standardised 90 min soccer match. Measurements were obtained at five time points (T0–T4): 24 h pre-match, 60 min pre-match, 10–15 min post-match, 24 h post-match, and 48 h post-match. At each time point, blood biomarkers, neuromuscular performance, sprint performance, maximal strength, and perceptual responses were assessed using standardised procedures.

Testing was organised around five time points: 24 h before the match (T0), 60 min before kick-off (T1), 10–15 min after the final whistle (T2), 24 h post-match (T3), and 48 h post-match (T4). At each time point, capillary blood samples were obtained first, followed by neuromuscular and perceptual measures in the same standardised order at each session to minimise measurement-order variability. Players were instructed to maintain their normal diet and sleep, to avoid strenuous exercise outside team training, and to abstain from alcohol and caffeine for 24 h before T0 and between T2 and T4 (Figure 1).

### 2.3. Capillary Blood Sampling and Handling

Capillary samples were collected using BD Microtainer^®^ Contact-Activated Lancets (BD, Franklin Lakes, NJ, USA) and BD Microtainer^®^ SST™ tubes suitable for low-volume capillary sampling. This low-volume approach was selected because it enables repeated field-based sampling with minimal disruption to athletes, which is advantageous when monitoring recovery across closely spaced time points. In the present study, capillary sampling was used to improve operational feasibility rather than to demonstrate methodological superiority over venous sampling [12,14,15,16]. Because capillary and venous concentrations may differ for some analytes owing to sampling site, local tissue fluid contribution, peripheral blood flow conditions, and pre-analytical variability, the present biomarker values should not be assumed to be directly interchangeable with venous reference values. Samples were centrifuged at 3000× *g* for 10 min at 4 °C using a Hettich EBA 200 refrigerated microcentrifuge (Hettich GmbH, Tuttlingen, Germany). Plasma aliquots were stored at −80 °C in Eppendorf 1.5 mL cryovials (Eppendorf, Hamburg, Germany) using an ultra-low temperature freezer (Thermo Scientific™ Forma™ 900 Series, Thermo Fisher Scientific, Waltham, MA, USA) until analysis [1,17].

### 2.4. Biochemical Analyses

Biochemical markers were intentionally selected to represent three complementary domains—muscle damage and repair, inflammatory–immune load, and endocrine–metabolic status—so that recovery could be evaluated as a multidimensional rather than single-marker phenomenon.

Muscle damage and repair markers comprised creatine kinase (CK), lactate dehydrogenase (LDH), myoglobin, urea, creatinine and irisin. These analytes were selected because they capture structural muscle strain, membrane disruption, protein turnover, and selected recovery-related myokine responses following high-intensity intermittent exercise. CK, LDH, urea and creatinine were analysed using enzymatic colourimetric assays on a Beckman Coulter AU480 Automated Chemistry Analyzer (Beckman Coulter, Brea, CA, USA). Myoglobin and irisin concentrations were measured using commercial ELISA kits and read on a BioTek Synergy HTX Multi-Mode Microplate Reader (Agilent BioTek, Winooski, VT, USA). All assays were performed in duplicate, and internal quality controls were run with each batch. Muscle damage marker measurements followed protocols validated for capillary sampling in exercise settings [14,18,19].

Inflammatory–immune markers included interleukin-6 (IL-6), tumour necrosis factor-α (TNF-α), high-sensitivity C-reactive protein (hsCRP), total white blood cell (WBC) count and leukocyte differential counts (neutrophils, lymphocytes). These measures were chosen to characterise both the immediate cytokine response and the subsequent systemic immune–inflammatory phase, thereby complementing the muscle damage profile rather than duplicating it. IL-6 and TNF-α were quantified using high-sensitivity ELISA kits (R&D Systems, Minneapolis, MN, USA) following the manufacturer’s protocols; hsCRP was analysed using a high-sensitivity immunoturbidimetric assay on the Roche Cobas c311 Analyzer (Roche Diagnostics, Mannheim, Germany). WBC and leukocyte differential counts were obtained using an automated haematology analyser (Sysmex XN-1000, Sysmex Corp., Kobe, Japan).

Endocrine–metabolic markers included serum cortisol, total testosterone, the testosterone/cortisol (T:C) ratio and blood lactate. These variables were included to reflect acute stress, anabolic–catabolic balance, and metabolic load, which may recover on a different timeline from inflammatory or mechanical markers. Cortisol and total testosterone concentrations were measured using chemiluminescent immunoassays on the Abbott Architect i2000SR system (Abbott Laboratories, Abbott Park, IL, USA). The T:C ratio was calculated from these values. Capillary blood lactate was analysed using a Lactate Pro 2 analyser (Arkray Inc., Kyoto, Japan) with compatible test strips, a device widely used in high-performance sport due to its strong agreement with laboratory enzymatic methods.

All biochemical analyses were performed in the same accredited laboratory, with the same equipment and technicians, and with coefficients of variation within the ranges recommended by the manufacturers. Inflammatory–immune and endocrine analyses used high-sensitivity assays and strictly standardised pre-analytical handling to optimise reliability given known capillary–venous differences [15,20]. The combined panel, together with capillary microsampling, was intended to provide a feasible descriptive approach for field-based characterisation of different biological layers of post-match fatigue and recovery, rather than to establish diagnostic cut-offs or validate a readiness model.

### 2.5. Neuromuscular Performance Tests

Neuromuscular performance was assessed via countermovement jump (CMJ), 20 m sprint and maximal voluntary contraction (MVC) of the knee extensors. These tests were selected because they reflect complementary aspects of post-match readiness, including explosive power, acceleration ability, and maximal force production, which may not track biochemical recovery in a uniform manner. All tests were conducted indoors or on a consistent surface, at the same time of day as the match, where possible [21].

CMJ height was assessed using a Just Jump System jump mat (Probotics Inc., Huntsville, AL, USA), which has demonstrated high reliability in elite athlete monitoring [21]. After a standardised warm-up, players completed three maximal CMJ trials separated by 30 s of rest, with the best performance retained for analysis.

Sprint performance over 20 m was recorded using Brower Timing Systems speed gates (Brower Timing Systems, Draper, UT, USA) placed at 0 and 20 m. After familiarisation and warm-up, each player performed three maximal 20 m sprints from a standing start, separated by standardised rest intervals. The fastest 20 m time was used for analysis.

Isometric knee extensor MVC was measured using a Biodex System 4 Pro isokinetic dynamometer (Biodex Medical Systems, Shirley, NY, USA), with the knee fixed at 60° of flexion. All assessments were preceded by the same standardised warm-up and were performed by the same technician at all time points.

### 2.6. Perceptual Measures

Delayed onset muscle soreness (DOMS) was assessed using a numerical rating scale (0–10) [22], where 0 represented ‘no soreness’, and 10 represented ‘extreme soreness.’ Players were instructed to rate overall lower limb muscle soreness during functional movements such as walking and squatting, consistent with previous research on exercise-induced muscle damage. Numerical rating scales have demonstrated acceptable reliability for DOMS assessment in athletic populations. DOMS was included because perceptual fatigue captures the athlete’s subjective experience of recovery and may remain disturbed even when selected biochemical or performance markers appear to improve.

All perceptual ratings were collected individually in a quiet environment to minimise peer influence and ensure independent reporting. Perceptual measures were treated as complementary descriptive information rather than as stand-alone indicators of physiological recovery.

### 2.7. Statistical Analysis

Given the fixed sample size inherent to elite team settings, a repeated-measures design and linear mixed-effects modelling were employed to account for within-player dependence. No formal a priori sample size calculation was undertaken; therefore, the inferential results should be interpreted cautiously, and this study should be regarded as descriptive and hypothesis-generating. All statistical analyses were conducted in R (version 4.5.0; R Foundation for Statistical Computing, Vienna, Austria) using the lme4, lmerTest, emmeans, performance, tidyverse, and rstatix packages. Data are presented as mean ± SD unless otherwise stated. Distributions were examined visually and using Shapiro–Wilk tests. For approximately normal variables, linear mixed-effects models were fitted with time (T0-T4) as a fixed effect and subject as a random intercept. Markedly skewed biomarkers were log10-transformed; if residuals remained non-normal, non-parametric procedures (Friedman tests with Wilcoxon signed-rank post hoc tests) were applied [12,23]. When a significant main effect of time was detected, post hoc comparisons were performed using T0 as the primary reference time point, with additional contrasts between post-match time points where relevant; *p* values were adjusted using the Benjamini–Hochberg false discovery rate procedure. Within-subject effect sizes were expressed as standardised mean differences with bootstrapped 95% confidence intervals. Because this study was observational and lacked a control condition, these analyses were intended to describe and compare time-course responses rather than to support causal inference.

Exploratory associations between peak biochemical responses (or percentage changes from baseline) and the largest decrements in performance or DOMS were assessed using Spearman rank correlations. These analyses were explicitly exploratory and hypothesis-generating and were not interpreted as evidence of causal, predictive, or mechanistic relationships, and no inference regarding directionality or underlying mechanisms can be drawn from these analyses. Related visualisations were used for descriptive purposes only and were not intended as confirmatory network or predictive modelling analyses. Statistical significance was accepted at *p* < 0.05.

To aid interpretation, outcomes were organised into three inter-related domains: (i) muscle damage and repair, (ii) inflammatory–immune and endocrine load, and (iii) neuromuscular–perceptual responses [24].

## 3. Results

### 3.1. Participants and Baseline Characteristics

Twenty-two elite male outfield players completed all testing sessions and were included in the analyses. Players were in their early twenties, with anthropometric characteristics (height, body mass, and body fat percentage) consistent with high-level soccer populations. Training history and maximal oxygen uptake values indicated a well-trained cohort (Table 1).

### 3.2. Muscle Damage and Repair

**Figure 2:**
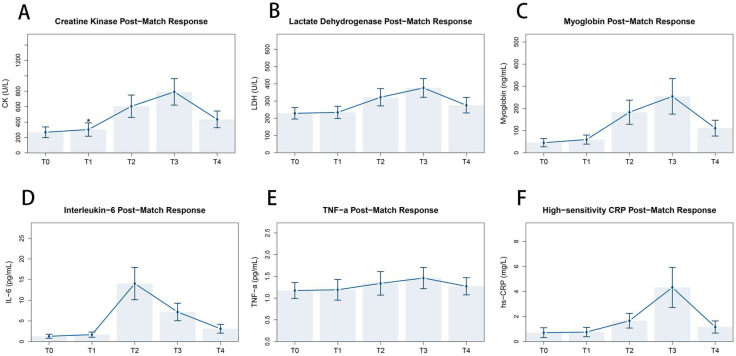
Time-course responses of capillary plasma markers following a standardised 90 min soccer match. (**A**) Creatine kinase (CK), (**B**) lactate dehydrogenase (LDH), (**C**) myoglobin, (**D**) interleukin-6 (IL-6), (**E**) tumour necrosis factor-α (TNF-α), and (**F**) high-sensitivity C-reactive protein (hs-CRP) across five time points: 24 h pre-match (T0), 60 min pre-match (T1), 10–15 min post-match (T2), 24 h post-match (T3), and 48 h post-match (T4). Data are presented as mean ± SD. CK, LDH, and myoglobin exhibited delayed peaks at 24 h (T3), whereas IL-6 showed a rapid acute surge immediately post-match (T2), followed by a return toward baseline. hs-CRP demonstrated a characteristic delayed inflammatory peak at T3. Asterisks denote significant differences vs. T0.

A clear asynchronous pattern was observed across domains. Inflammatory and endocrine disturbances were most evident immediately after match play, classical muscle damage markers peaked during early recovery, and neuromuscular–perceptual outcomes showed both immediate and delayed impairments. Match play elicited marked elevations in indices of skeletal muscle damage and repair (Table 2; Figure 2A–C). CK increased after the match, peaked at 24 h, and remained above baseline at 48 h.

LDH and myoglobin followed similar delayed kinetics, with peak values observed at 24 h and partial recovery by 48 h. These results indicate a delayed muscle damage profile rather than a uniformly immediate post-match response.

**Figure 3:**
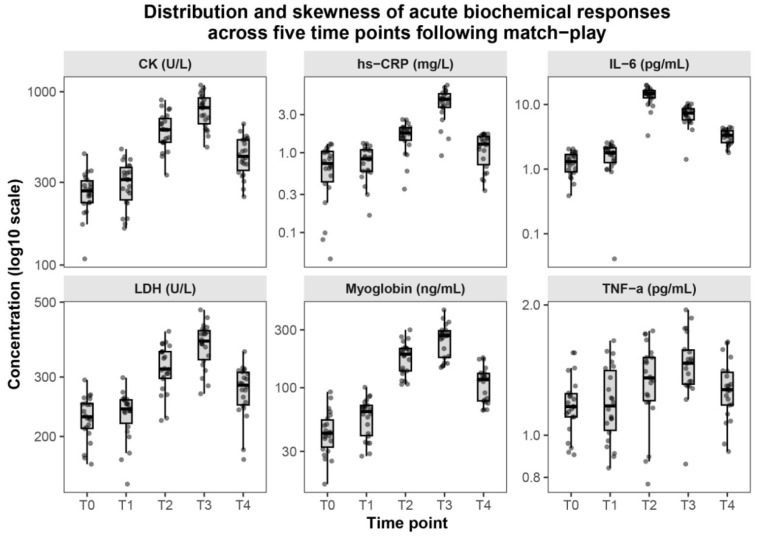
Distribution and skewness of acute biochemical responses across five time points. Box-and-whisker plots illustrating the distribution, variability, and right-skewed characteristics of six capillary biochemical markers across the five measurement time points: 24 h pre-match (T0), 60 min pre-match (T1), 10–15 min post-match (T2), 24 h post-match (T3), and 48 h post-match (T4). Concentrations are displayed on a log10 scale to accommodate substantial inter-individual variability typical of exercise-induced biomarker responses. Markers include creatine kinase (CK), lactate dehydrogenase (LDH), myoglobin, interleukin-6 (IL-6), tumour necrosis factor-α (TNF-α), and high-sensitivity C-reactive protein (hs-CRP). The plots highlight the large right-tail distribution for CK, myoglobin, and IL-6—indicative of heterogeneous muscle damage and acute cytokine release—alongside the delayed, markedly skewed response pattern observed for hs-CRP at 24 h post-match.

**Figure 4:**
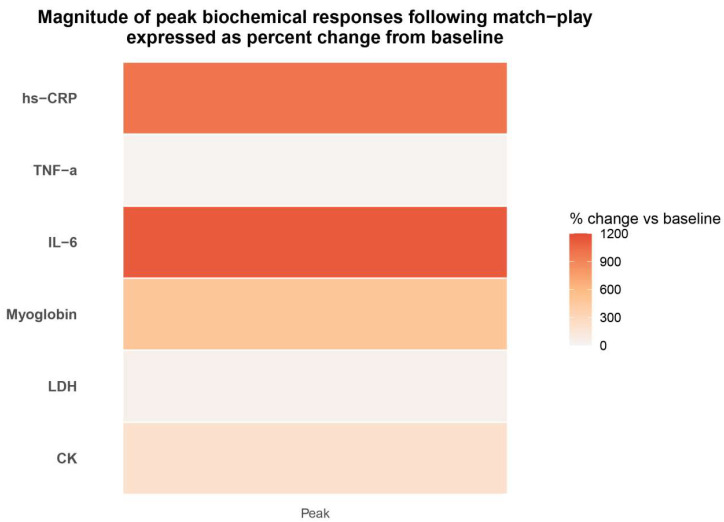
Peak biochemical responses expressed as percent change from baseline. Heatmap illustrating the relative magnitude of peak changes (% from T0) for CK, LDH, myoglobin, IL-6, TNF-α and hs-CRP. Warmer colours indicate larger responses.

These kinetics are summarised in Figure 2A–C; skewed distributions and inter-individual variability are shown on a log10 scale in Figure 3, and peak percentage changes are visualised in Figure 4.

Systemic markers related to protein turnover and renal handling showed a parallel, though more moderate, pattern (Table 2). Urea and creatinine increased after the match, were highest during early recovery, and then moved back toward baseline by 48 h. Together, these changes are consistent with transient protein turnover and renal handling responses during early recovery.

Irisin increased immediately post-match and approached baseline by 48 h. This transient myokine response contrasted with the more delayed peak observed in CK, LDH, and myoglobin.

**Figure 5:**
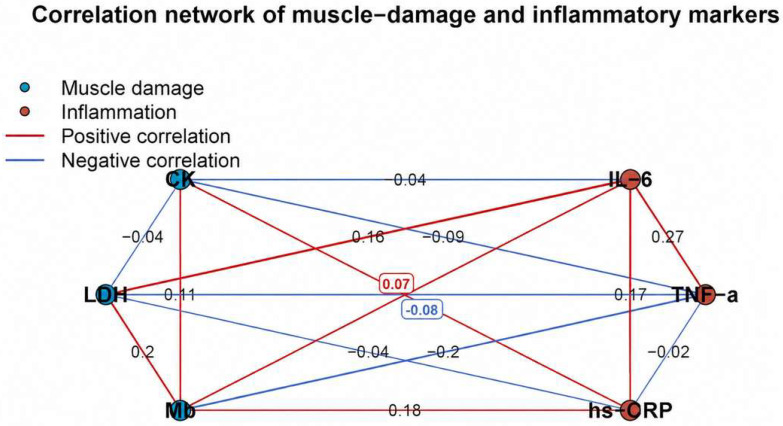
Exploratory correlation network linking muscle damage and inflammatory markers. Network plot showing positive (red) and negative (blue) Spearman correlations between CK, LDH, myoglobin, IL-6, TNF-alpha, and hsCRP. Nodes are grouped by marker type. This visualisation is descriptive and should not be interpreted as evidence of causal or mechanistic relationships.

Raw individual-level biochemical values at all time points are provided in Supplementary Table S1, with full trajectories in Figure S1, raincloud distributions in Figure S2, and extended heatmaps in Figure S3. These supplements highlight inter-individual heterogeneity while supporting a coherent delayed onset damage profile. The exploratory correlation structure among the key biomarkers is presented in Figure 5.

### 3.3. Inflammatory–Immune and Endocrine Load

Inflammatory–immune and endocrine variables showed a more rapid response profile than the classical muscle damage markers. IL-6 increased sharply immediately post-match (T2; *** *p* < 0.001) and then declined toward baseline by 24–48 h, whereas hsCRP displayed a delayed peak at 24 h (T3) and remained above baseline at 48 h. TNF-α changed only modestly across time points (Table 3; Figure 2D–F).

**Figure 6:**
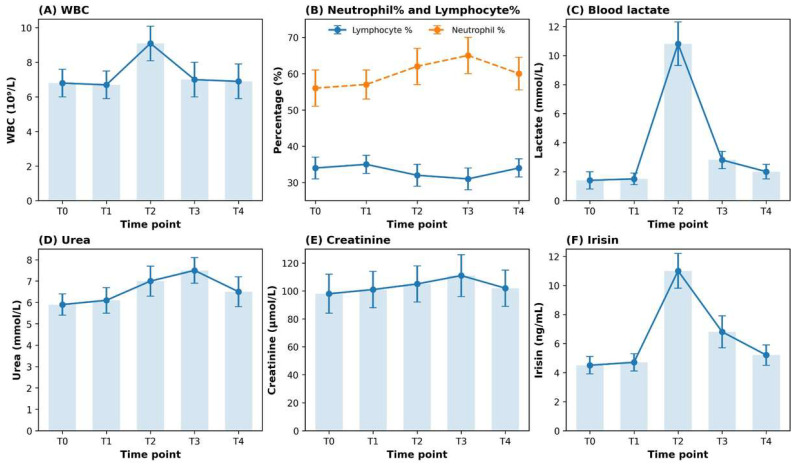
Time-course responses of immune and metabolic markers across five time points. Mean (±SD) values for (**A**) white blood cell count (WBC), (**B**) neutrophil and lymphocyte percentages, (**C**) blood lactate, (**D**) urea, (**E**) creatinine and (**F**) irisin. Shaded bars represent relative changes from baseline.

**Figure 7:**
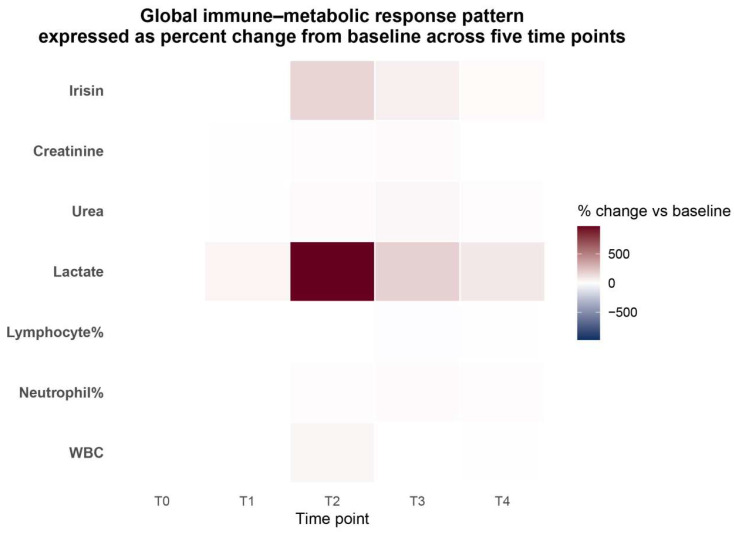
Global immune–metabolic response pattern expressed as percent change from baseline. Heatmap summarising relative (% from T0) deviations across lactate, urea, creatinine, irisin, neutrophil percentage, lymphocyte percentage and WBC from T0 to T4.

Leukocyte counts mirrored the acute inflammatory response. WBC increased significantly immediately post-match and remained mildly elevated during early recovery. Lymphocyte percentage decreased modestly at T2 (32.0 ± 3.8% vs. 34.2 ± 4.3% at T0, ** *p* < 0.01) and remained slightly reduced at T3 before normalising by T4 (Table 3; Figure 6A,B and Figure 7). Neutrophil percentage showed the complementary upward pattern, consistent with a transient post-match immune shift.

Endocrine markers likewise showed an acute, time-limited stress response. Cortisol increased significantly immediately post-match (*** *p* < 0.001) and returned toward baseline by 24–48 h. Total testosterone decreased transiently after the match, rebounded above baseline at 24 h, and remained stable at 48 h. Consequently, the T:C ratio decreased significantly immediately post-match and recovered by 24–48 h, indicating a short-lived catabolic shift rather than prolonged endocrine disruption (Table 3).

**Figure 8:**
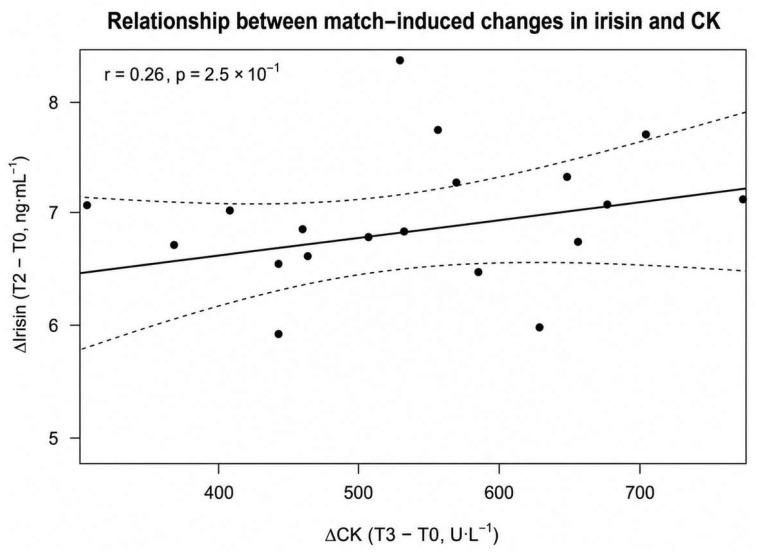
Exploratory association between match-induced changes in irisin and CK. Scatter plot showing the relationship between Δirisin (T2-T0) and ΔCK (T3-T0), with fitted regression line and 95% confidence interval. The plot is intended for descriptive visualisation only.

**Figure 9:**
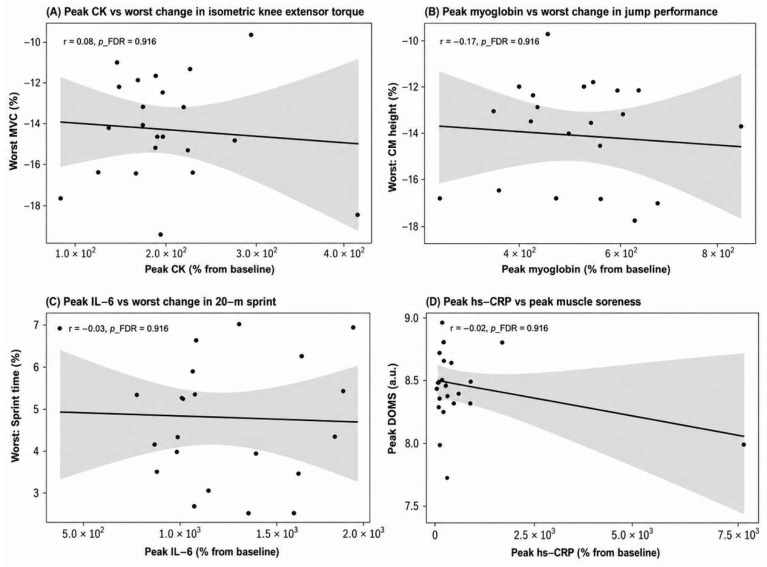
Exploratory associations between peak biochemical responses and worst neuromuscular or perceptual changes. Scatter plots with fitted regression lines for (**A**) peak CK vs. worst change in MVC, (**B**) peak myoglobin vs. worst change in CMJ height, (**C**) peak IL-6 vs. worst change in 20 m sprint time, and (**D**) peak hsCRP vs. peak DOMS. Shaded areas indicate 95% confidence intervals. These exploratory associations should not be interpreted as causal or predictive evidence. These associations are exploratory and should not be interpreted as causal, predictive, or mechanistic relationships.

The combined immune–metabolic response pattern across WBC, leukocyte differentials, lactate, urea, creatinine and irisin is summarised in Figure 6 and Figure 7. Illustrative exploratory associations among selected biomarkers and between biomarkers and functional outcomes are shown in Figure 8 and Figure 9.

### 3.4. Neuromuscular–Perceptual Recovery

**Figure 10:**
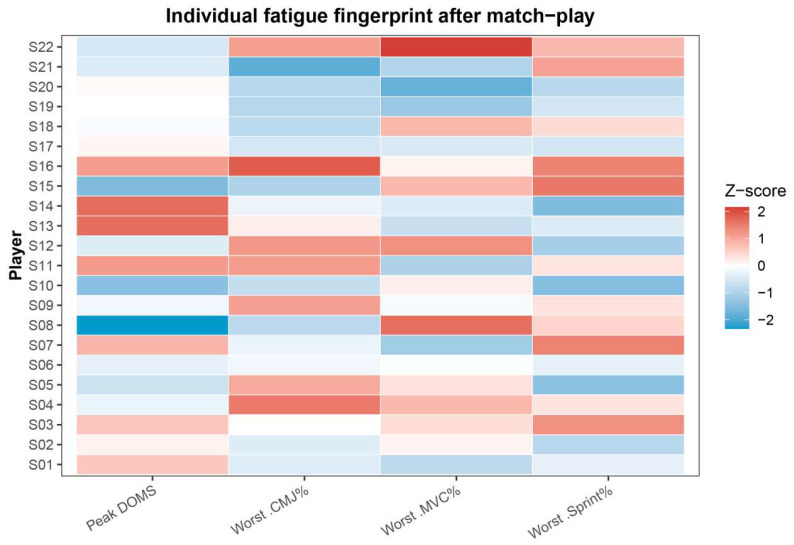
Individual fatigue fingerprint following match play. Z-score heatmap displaying inter-individual variability in peak DOMS, worst CMJ%, worst MVC% and worst sprint% across the 22 players.

**Figure 11:**
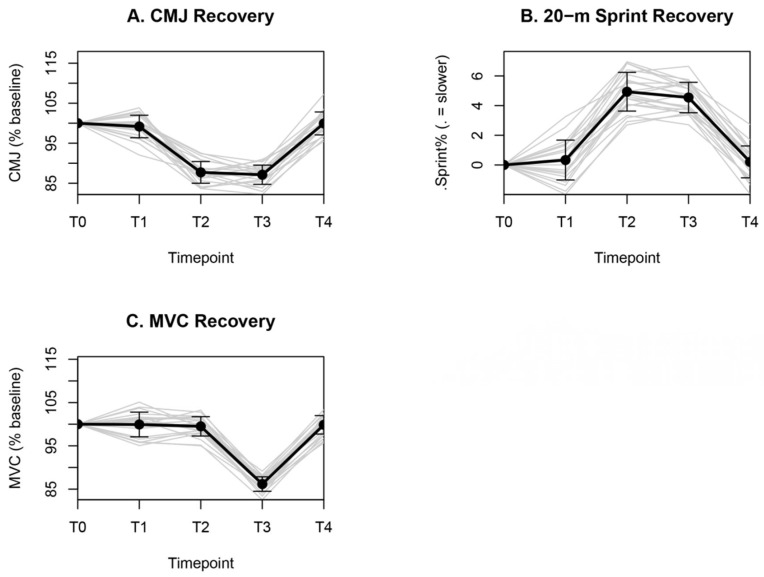
Neuromuscular and perceptual recovery across 48 h post-match. Mean (±SD) and individual trajectories for (**A**) CMJ height, (**B**) 20 m sprint time and (**C**) MVC from T0 to T4.

Neuromuscular performance and perceptual outcomes also demonstrated asynchronous recovery, but their pattern differed from the inflammatory and endocrine responses (Table 4, Figure 6C, Figure 10 and Figure 11). Blood lactate increased markedly immediately post-match and returned toward baseline during recovery, consistent with a transient increase in glycolytic flux and lactate turnover during match play rather than a delayed recovery impairment.

CMJ height declined significantly immediately post-match, remained suppressed at 24 h, and returned to baseline by 48 h.

Sprint time increased post-match, with the greatest delay observed immediately after the match and residual impairment still evident at 24 h; performance returned to baseline by 48 h.

Maximal knee strength showed a different trajectory: MVC was relatively preserved immediately post-match but decreased significantly at 24 h before returning to baseline by 48 h (Figure 11C). Thus, maximal strength exhibited its greatest decrement during early recovery rather than immediately after the match.

Perceptual markers broadly paralleled the delayed muscle damage profile. DOMS increased significantly post-match, peaked at 24 h, and remained elevated at 48 h.

Overall, explosive outcomes (CMJ and sprint) recovered to baseline by 48 h, whereas maximal strength and soreness showed greater delayed impairment. This pattern reinforces that apparent recovery in one functional test does not necessarily indicate complete recovery across other physiological or perceptual domains.

### 3.5. Exploratory Associations Between Biomarkers, Performance and Soreness

Exploratory analyses were undertaken to examine descriptively whether biochemical responses tended to co-vary with subsequent alterations in neuromuscular performance and perceptual soreness. These analyses were not used to infer causality but rather to visualise the extent to which selected markers showed aligned or dissociated responses across domains.

As illustrated in Figure 5, positive associations were observed among CK, LDH, and myoglobin, indicating concordant variation among these classical indices of muscle damage within the present sample. Moderate correlations were also observed between muscle damage markers and inflammatory mediators such as hsCRP and IL-6, indicating a pattern of concurrent variation between circulating damage-related enzymes and inflammatory markers within the present dataset.

Further exploratory analyses showed that larger myoglobin and LDH responses were observed to co-occur with greater reductions in CMJ performance, whereas larger CK and myoglobin responses tended to coincide with higher DOMS severity. These observations remain descriptive and do not establish causal pathways, mechanistic direction, or predictive validity.

Overall, these visualisations suggest that post-match responses span multiple physiological domains and are only partly aligned in their time course and magnitude. The network analysis should therefore be interpreted as descriptive and hypothesis-generating only, not as definitive evidence of mechanistic interdependence or a fixed systems hierarchy.

### 3.6. Individual Fatigue Profiles

To visualise inter-individual variability in the neuromuscular–perceptual response, fatigue fingerprints combining peak DOMS and worst relative changes in CMJ height, MVC and 20 m sprint time were constructed for each player (Figure 10). The Z-score heatmap shows marked heterogeneity (Figure 10): some players exhibited large decrements in jump and sprint performance with relatively modest soreness, whereas others showed pronounced DOMS with smaller performance impairments. A subset of players displayed consistently high Z-scores across all four dimensions, indicative of a globally heavier fatigue burden.

These individual profiles highlight that players with similar group mean responses can nonetheless follow distinct recovery trajectories across different outcome domains. From an applied perspective, this illustrates the potential usefulness of combining objective performance tests with soreness ratings while also emphasising that such profiles are descriptive and require validation before being used as formal decision tools.

An extended version of these fingerprints, incorporating additional performance and biochemical variables, is provided in Figure S7, which offers a more detailed view of player-specific fatigue patterns across the 48 h recovery period.

### 3.7. Descriptive Domain-Level Recovery Profile

**Figure 12:**
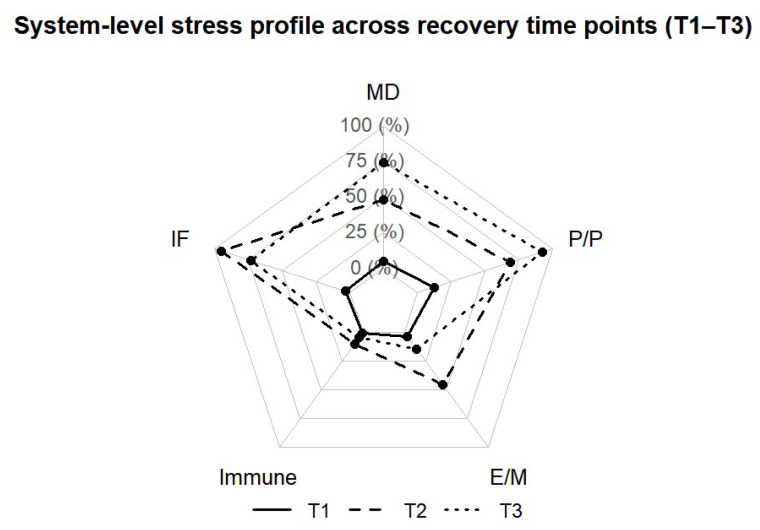
Descriptive domain-level recovery profile across recovery time points. Radar plot showing normalised deviations from baseline across five descriptive domains: muscle damage (MD), inflammatory response (IF), immune function (Immune), endocrine–metabolic load (E/M), and performance/perceptual outcomes (P/P) at T1–T3. Higher values indicate greater observed perturbation. The radar plot summarises asynchronous recovery patterns but does not represent a validated readiness model or decision-making tool.

To provide a compact descriptive overview of the temporal pattern of post-match perturbation, standardised domain-level summaries were visualised using a radar plot (Figure 12). The plot illustrates that different domains contributed unequally across time points: inflammatory and endocrine responses were most pronounced immediately after the match, whereas muscle damage indices, strength loss, and soreness were more evident during early recovery. Rather than constituting a formal systems model, this visualisation was intended to summarise the practical value of integrated multi-domain monitoring across the 48 h window.

At T1 (60 min pre-match), all domains were close to baseline, indicating that players commenced the match in a relatively recovered state. Immediately post-match (T2), the profile was dominated by elevations in IF, Immune and E/M, alongside clear but more modest perturbations in the P/P domain. This configuration reflects an acute phase characterised by inflammatory–immune activation, endocrine–metabolic stress and substantial internal stress, with early decrements in explosive performance. Because external load data were unavailable, this pattern cannot be linked to quantified running or acceleration-deceleration demands.

By 24 h post-match (T3), the shape of the radar plot shifted. IF, Immune and E/M domains had moved back towards baseline, whereas MD and P/P showed their greatest deviations. Muscle damage markers were at or near their peak, and the P/P domain remained markedly perturbed due to the combination of strength deficits and maximal DOMS, despite partial recovery of jump and sprint performance. This pattern indicates that the early recovery phase is dominated by structural muscle stress and soreness rather than ongoing systemic inflammatory or endocrine strain.

Taken together, the descriptive domain-level profile underscores a temporal dissociation between domains: acute inflammatory–immune and endocrine perturbations resolve relatively quickly, whereas muscle damage and perceptual recovery lag behind. From an applied perspective, the radar representation provides a compact visual overview of how different physiological systems contribute to the overall fatigue burden at each time point, but it should not be interpreted as a validated readiness model or decision-making tool. The full time-course panels for the biochemical and performance variables underlying these domain summaries are provided in Supplementary Figure S6.

Radar plot summarising relative changes across five physiological domains—muscle damage (MD), inflammatory markers (IF), immune markers (Immune), endocrine–metabolic markers (E/M) and performance/perceptual measures (P/P)—at T1, T2 and T3. Circles, triangles and squares represent the three time points, with dashed lines illustrating the overall profile at each stage of recovery. The plot is descriptive and does not represent a validated readiness algorithm.

## 4. Discussion

### 4.1. Findings

The present study provides a field-based, time-resolved description of biochemical, neuromuscular, and perceptual responses during the first 48 h after a standardised soccer match in elite players. The principal finding was that recovery did not occur uniformly across physiological systems. Instead, inflammatory–endocrine responses were most pronounced immediately after match play, whereas muscle damage markers, maximal strength impairment, and perceived soreness showed their greatest disturbances during the subsequent recovery period. Together, these observations support the concept that post-match recovery is multidimensional and temporally asynchronous rather than a single linear return to baseline [2,9,11].

The overall response profile observed in the present study was broadly consistent with the previous literature describing acute and residual fatigue after soccer match play. Immediate elevations in IL-6, cortisol, leukocyte responses, and blood lactate were accompanied by delayed increases in CK, LDH, myoglobin, hsCRP, DOMS, and maximal force impairment. In contrast, CMJ and sprint performance showed substantial acute decrements but recovered more rapidly than some markers of tissue-level stress. These findings indicate that different physiological systems may recover on partially independent timelines following high-intensity intermittent competition.

From an applied perspective, the present data reinforce the importance of interpreting post-match fatigue using multiple complementary domains rather than relying on a single biomarker or performance measure. The rapid normalisation of some variables despite persistent disturbances in others suggests that apparent recovery in one domain may not necessarily indicate complete restoration of physiological function. Collectively, the findings support the practical value of integrated monitoring approaches capable of capturing both acute systemic stress and delayed tissue-level recovery processes within elite soccer environments.

The broader physiological interpretation of these asynchronous responses, together with their potential mechanistic basis and relationship to the existing literature, is discussed in the following sections.

### 4.2. Physiological Interpretation and Comparison with Previous Literature

Taken together, these findings suggest that post-match fatigue should be understood as an integrated, multi-system response in which inflammatory, metabolic, and neuromuscular processes evolve on partially overlapping but non-synchronous timelines. The asynchronous recovery profile observed across domains likely reflects the fact that different physiological systems are constrained by different recovery bottlenecks at different stages following match play.

The asynchronous recovery profile observed across systems likely reflects fundamental physiological differences in how match-induced stress is initiated, expressed, and resolved. Inflammatory–endocrine responses are driven primarily by acute metabolic, neuroendocrine, and immune activation and therefore tend to occur immediately after competition and normalise relatively quickly [25,26,27]. By contrast, CK, LDH, myoglobin, DOMS, and delayed strength loss are downstream manifestations of structural muscle stress, excitation–contraction disturbance, nociceptive sensitisation, and repair processes, which generally become most evident during the early recovery window [18,28,29].

Viewed physiologically, the recovery profile suggests a shift from an acute systemic phase to a delayed tissue-level phase. Immediately after the match, the prominent lactate, IL-6, cortisol and leukocyte responses most likely reflected high glycolytic flux, sympathetic adrenal activation and transient immune cell mobilisation. By 24 h, the dominant signal had shifted toward muscle membrane disruption, local inflammatory signalling, nociceptive input and reduced voluntary force production, as indicated by the delayed peaks in CK, LDH, myoglobin, hsCRP and DOMS, together with the MVC nadir. Such sequencing is consistent with evidence that exercise-induced cytokine and endocrine responses can occur rapidly, whereas structural muscle damage, inflammatory remodelling and soreness-related functional impairment usually unfold over a longer recovery window [30,31,32,33]. This helps explain why some markers were already moving back toward baseline while others were still close to their greatest disturbance.

In the present cohort, IL-6 rose sharply immediately post-match, whereas hsCRP peaked later at 24 h, reproducing the established distinction between rapid cytokine signalling and delayed hepatic inflammatory responses [17,29,34,35]. Cortisol and the T:C ratio also showed the expected acute stress profile, with substantial immediate perturbation followed by recovery toward baseline within 24–48 h [14,15,36]. These findings support the view that inflammatory–endocrine markers are informative for identifying the immediate internal stress imposed by match play but may be less useful as isolated indicators of ongoing recovery status later in the microcycle.

Blood lactate should also be interpreted within this dynamic framework. The post-match rise in lactate is better viewed as a marker of rapid glycolytic flux and lactate turnover during intense intermittent work rather than as a metabolic waste product or a direct marker of fatigue. Contemporary lactate shuttle theory indicates that lactate can act as an oxidisable substrate, a gluconeogenic precursor and a signalling molecule, moving between producer and consumer tissues according to concentration gradients and mitochondrial oxidative demand [37,38,39,40,41]. This broader interpretation is important because lactate may participate in redox regulation, mitochondrial adaptation, angiogenic signalling and muscle–cell communication rather than simply accumulating as an endpoint of anaerobic metabolism. Therefore, the rapid rise and decline of lactate in the present study most likely reflects acute match-induced metabolic exchange and clearance, whereas the later disturbances in CK, hsCRP, MVC and DOMS reflect slower tissue-level and neuromuscular recovery. The rapid normalisation of lactate despite persistent disturbances in CK, MVC and DOMS further supports the concept that metabolic recovery and tissue-level recovery represent partially independent dimensions of post-exercise restoration.

By contrast, the delayed peak of CK, LDH, and myoglobin at 24 h, together with the nadir in MVC and the peak in DOMS, points to an early recovery phase dominated by tissue-level muscle stress rather than by continuing acute endocrine or cytokine disturbance [29,42,43]. This delayed profile is consistent with patterns previously attributed to membrane disruption and muscle repair processes in the literature [2,3,9].

The timing of IL-6 and hsCRP further supports this interpretation. IL-6 can rise rapidly during and after intense intermittent exercise and may act partly as a muscle-derived cytokine or myokine, whereas CRP is produced mainly downstream of cytokine signalling and therefore tends to peak later. In addition to its inflammatory signalling role, exercise-induced IL-6 may also contribute to substrate mobilisation and metabolic regulation during prolonged intermittent exercise. In this sense, the early cytokine response and the later hsCRP response should not be treated as identical inflammatory events; rather, they represent linked but temporally distinct layers of the post-match inflammatory process [30,32,33,44].

The neuromuscular–perceptual findings reinforce this interpretation while also requiring careful differentiation between outcome types. CMJ and sprint performance were impaired after the match but largely recovered by 48 h, whereas maximal force production showed its largest decrement at 24 h, and DOMS remained elevated at 48 h. DOMS should not be interpreted as a direct or exclusive marker of structural muscle damage because soreness is influenced by inflammatory mediators, nociceptor sensitisation, connective tissue stress, movement-related discomfort, and central perception. Similarly, CMJ, sprint, and MVC assess different aspects of neuromuscular function and should not be treated as interchangeable readiness indicators [24,45].

In this context, DOMS, CMJ, sprint performance and MVC should be read as overlapping but not equivalent windows into recovery. DOMS captures a perceptual pain response influenced by nociceptor sensitisation, connective tissue loading and central appraisal; CMJ and sprint performance depend on rapid force production, stretch-shortening cycle function and intermuscular coordination; MVC is more directly constrained by maximal voluntary drive and peripheral force-generating capacity. This interpretation is consistent with the exercise-induced muscle damage literature, showing that soreness, force loss and explosive performance can recover on different timelines after damaging or eccentric-biased exercise [31,46,47,48]. Their partial dissociation in the present study therefore has physiological meaning rather than representing measurement inconsistency.

The biochemical time courses observed in this study align with, yet refine, existing descriptions of post-match fatigue in elite soccer players [49]. The magnitude and timing of CK, LDH, and myoglobin responses fall within the range reported after competitive matches, with CK typically rising 2–5 fold and peaking approximately 18–30 h after play [50]. Our data therefore support 24 h as a critical window for detecting residual structural muscle stress in well-trained soccer players, in contrast to later peaks sometimes seen after isolated eccentric or unaccustomed exercise [51].

The inflammatory–immune responses were also consistent with previous reports. The rapid IL-6 surge, modest TNF-α change, delayed hsCRP increase, and transient leukocyte redistribution mirror the kinetics reported in official and simulated match play studies [34,35,52]. Together with the short-lived endocrine perturbation, these data reinforce that acute internal stress and later recovery impairment are related but not identical phenomena.

The endocrine responses likewise followed the expected short-term profile reported in elite athletes, with an acute rise in cortisol and transient suppression of the T:C ratio followed by recovery over the subsequent 24–48 h [36]. This pattern supports the interpretation that endocrine variables were most useful for capturing immediate post-match strain rather than prolonged unresolved fatigue in the present context.

The neuromuscular findings corroborate earlier work showing that CMJ and sprint impairments are most pronounced immediately after match play and tend to recover within 24–48 h, whereas maximal strength may show a delayed decrement [53,54]. From a physiological perspective, immediate decrements in CMJ and sprint may reflect acute metabolic fatigue, transient reductions in neural drive, and altered excitation–contraction coupling, whereas delayed MVC impairment may reflect a greater contribution of peripheral muscle dysfunction, soreness-related inhibition, and incomplete recovery of force-generating capacity. The co-occurrence of peak DOMS and peak CK at 24 h further suggests that subjective soreness tracked the delayed muscle damage phase more closely than the acute cytokine response.

An applied strength of the present study lies in the field-based integration of capillary biomarkers with neuromuscular and perceptual measures across closely spaced recovery time points within an elite team environment. Rather than establishing a new monitoring framework, the data provide a practical illustration that different physiological domains can indicate different recovery states at the same time point. This applied perspective may be useful for practitioners, but it should be interpreted within the descriptive scope of the present design.

### 4.3. Neuromuscular Responses Within the Integrated Recovery Process

The neuromuscular performance impairments observed in the present study are best interpreted within the broader context of the multi-domain recovery profile. CMJ and sprint performance were most impaired immediately after match play and recovered substantially within 48 h, whereas MVC demonstrated a more delayed decrement during early recovery. This divergence suggests that practitioners should avoid treating any single field test as a global proxy for recovery or readiness status, as different neuromuscular outcomes may reflect distinct physiological components of post-match fatigue [24,53].

From a physiological perspective, the different recovery trajectories observed across CMJ, sprint performance, MVC, and DOMS likely reflect differences in the functional properties captured by each measure. Explosive tasks such as CMJ and sprint performance are influenced strongly by rapid force production, stretch-shortening cycle efficiency, intermuscular coordination, and transient neural–metabolic fatigue. By contrast, MVC may be more sensitive to delayed peripheral muscle dysfunction, soreness-related inhibition, and incomplete recovery of maximal force-generating capacity.

The distinction between central and peripheral contributors to fatigue may help explain these patterns. Immediate impairments in CMJ and sprint performance may involve transient reductions in neural drive, altered motor unit recruitment, excitation–contraction disturbances, and acute metabolic perturbations associated with intense intermittent exercise. The delayed reduction in MVC observed at 24 h is more consistent with a stronger peripheral contribution, including residual muscle damage, inflammatory sensitisation, and inhibitory afferent feedback arising from mechanically stressed or sore muscle tissue [46,55,56,57,58]. Such feedback may reduce voluntary activation and maximal force output even when selected explosive performance measures have already begun to recover.

Perceptual recovery should also be interpreted within this multidimensional framework. DOMS did not fully parallel either biochemical or neuromuscular recovery trajectories, supporting the view that soreness reflects a complex interaction among inflammatory signalling, connective tissue stress, nociceptor sensitisation, movement-related discomfort, and central perceptual processing rather than a direct measure of structural muscle damage alone. The persistence of elevated DOMS despite partial recovery in CMJ and sprint performance therefore further reinforces the concept that different recovery domains may remain dissociated across the post-match recovery period.

Taken together, the present neuromuscular findings support the broader interpretation that post-match fatigue is task-specific and physiologically multidimensional. Accordingly, integrated assessment approaches combining explosive performance, maximal strength, biochemical responses, and perceptual measures may provide a more informative description of recovery status than reliance on any isolated marker alone.

### 4.4. Practical Implications for Post-Match Monitoring

From an applied perspective, the present findings support a multi-domain interpretation of post-match recovery rather than reliance on any isolated biochemical, neuromuscular, or perceptual marker alone [10,51]. The asynchronous recovery patterns observed across inflammatory endocrine, muscle damage, neuromuscular, and perceptual domains suggest that different physiological systems may indicate different recovery states at the same time point following competition.

In practical terms, the immediate post-match phase appeared to be characterised predominantly by acute metabolic, inflammatory, and endocrine perturbations, whereas the subsequent 24 h recovery period was more strongly associated with tissue-level muscle stress, delayed strength impairment, and elevated soreness. Accordingly, assessments performed immediately after competition may provide useful information regarding acute internal stress but may not fully reflect unresolved recovery demands during the following recovery period.

The present findings also reinforce the importance of interpreting CMJ, sprint performance, MVC, DOMS, and biochemical markers as complementary rather than interchangeable indicators of recovery status. Explosive performance measures recovered more rapidly than maximal force capacity and soreness, indicating that apparent normalisation in one functional outcome should not necessarily be interpreted as complete physiological recovery across all domains.

Within elite soccer environments characterised by congested schedules and limited recovery windows, integrated monitoring approaches combining biochemical, neuromuscular, and perceptual information may therefore provide a more comprehensive description of post-match recovery dynamics than reliance on any single variable alone. However, these observations should be interpreted within the descriptive scope of the present study and should ideally be integrated with contextual information such as external load, internal load, sleep, wellness, and individual longitudinal baselines when applied in practice.

Overall, the present data support the practical concept that the dominant physiological constraints during recovery may shift over time, moving from acute systemic stress immediately after match play toward delayed tissue-level and force production disturbances during the subsequent recovery period.

### 4.5. Limitations and Future Directions

Several limitations should be considered when interpreting the present findings. First, this study was conducted within a single elite male soccer squad, and the sample size was constrained by player availability rather than a formal a priori power calculation. Although this design reflects the practical realities of applied elite team research and supports within-player longitudinal interpretation, the findings may not generalise directly to female players, youth athletes, lower competitive levels, or other team sport populations.

Second, the observational repeated-measures design did not include a non-match control condition. Accordingly, this study was able to characterise temporal recovery patterns following a standardised match exposure but could not isolate the specific causal contribution of match play from other contextual influences such as sleep, nutrition, psychological stress, circadian variation, or routine team activities.

Third, objective external and internal load metrics were unavailable. In the absence of GPS-derived running demands, accelerations and decelerations, sprint counts, player load, and heart rate responses, it was not possible to directly relate inter-individual variability in biochemical, neuromuscular, or perceptual responses to quantified workload characteristics. Similarly, environmental conditions were not continuously monitored or incorporated into the statistical analyses, and therefore, environmental influences on recovery responses cannot be excluded.

Fourth, the monitoring window was limited to 48 h and may not have captured the complete recovery trajectory of selected biomarkers or neuromuscular outcomes, particularly because CK, myoglobin, and DOMS remained elevated at the final time point. In addition, all blood measurements were obtained using capillary microsampling to maximise field feasibility; therefore, absolute biomarker concentrations should not be assumed to be directly interchangeable with venous reference values.

Finally, the correlation networks, radar plots, and individual fatigue fingerprints presented in this study were intended as descriptive visualisations rather than validated predictive or mechanistic models. Future research should incorporate larger and more diverse cohorts, quantified match load monitoring, longer follow-up periods, environmental tracking, and confirmatory study designs capable of examining recovery mechanisms and load-response relationships more directly.

Despite these limitations, the present study provides an integrated field-based description of biochemical, neuromuscular, and perceptual recovery responses following elite soccer match play and contributes to the growing literature highlighting the multidimensional and asynchronous nature of post-match recovery.

## 5. Conclusions

This observational study provides a field-based, time-resolved description of biochemical, neuromuscular, and perceptual responses during the first 48 h after a standardised 90 min soccer match in elite male players. The findings suggest that post-match recovery is multidimensional and asynchronous rather than uniform.

Inflammatory–immune and endocrine perturbations were most evident immediately after the match and recovered relatively quickly, whereas muscle damage indices, maximal strength loss, and soreness peaked during early recovery and were not fully resolved at 48 h despite recovery of CMJ and sprint performance. These findings indicate that apparent recovery in one domain should not be assumed to represent complete player readiness across all domains.

A capillary-based, multi-domain perspective may therefore provide a feasible descriptive option for repeated field-based observation of post-match fatigue and recovery. However, the present study does not validate a readiness model, decision-making algorithm, or causal recovery framework. Future work should incorporate quantified external and internal load, environmental monitoring, appropriate control conditions, broader samples, longer follow-up, and integrated readiness models before stronger applied recommendations can be made.

Collectively, the present findings support the concept that different physiological systems recover on partially independent timelines following elite soccer match play, reinforcing the importance of integrated multi-domain recovery assessment in applied high-performance environments.

## Figures and Tables

**Table 1:** Baseline characteristics of the participants (*n* = 22).

Variable	Mean ± SD
Age (years)	23.8 ± 2.0
Height (cm)	180.9 ± 3.6
Body mass (kg)	76.3 ± 4.1
BMI (kg/m^2^)	23.3 ± 1.1
Body fat (%)	11.1 ± 1.8
Training age (years)	10.0 ± 1.8
VO_2_max (ml·kg^−1^·min^−1^)	59.3 ± 3.4
HRmax (bpm)	195.7 ± 4.1

**Table 2:** Muscle damage and repair markers (mean ± SD across timepoints).

Variable	T0 (Pre)	T1 (60 Min Pre-Match)	T2 (Post-Match)	T3 (24 h)	T4 (48 h)
CK (U/L)	268.0 ± 69.3	303.2 ± 86.4	605.3 ± 145.6	791.9 ± 171.6	435.3 ± 108.6
LDH (U/L)	228.9 ± 33.2	234.2 ± 35.6	322.0 ± 50.2	375.8 ± 54.2	275.6 ± 44.8
Myoglobin (ng/mL)	45.5 ± 18.7	59.6 ± 20.5	183.0 ± 54.4	254.6 ± 80.1	111.1 ± 35.7
Urea (mmol/L)	5.9 ± 1.3	6.1 ± 1.2	7.1 ± 1.2	7.6 ± 1.1	6.5 ± 0.9
Creatinine (μmol/L)	97.3 ± 11.0	100.5 ± 12.7	105.5 ± 14.0	111.0 ± 15.3	102.0 ± 13.8
Irisin (ng/mL)	4.3 ± 1.3	4.5 ± 1.4	11.0 ± 3.8	6.8 ± 2.7	5.0 ± 1.8

**Table 3:** Inflammatory–immune and endocrine load markers (mean ± SD).

Variable	T0 (Pre)	T1 (60 Min Pre-Match)	T2 (Post-Match)	T3 (24 h)	T4 (48 h)
IL-6 (pg/mL)	1.3 ± 0.5	1.7 ± 0.6	14.0 ± 3.9	7.2 ± 2.1	3.1 ± 1.1
TNF-α (pg/mL)	1.2 ± 0.2	1.2 ± 0.2	1.3 ± 0.3	1.5 ± 0.2	1.3 ± 0.2
hsCRP (mg/L)	0.7 ± 0.4	0.8 ± 0.4	1.7 ± 0.6	4.3 ± 1.6	1.2 ± 0.5
WBC (10^9^/L)	6.83 ± 1.51	6.73 ± 1.45	9.12 ± 1.89	7.10 ± 1.25	6.96 ± 1.16
Neutrophils (%)	56.5 ± 5.6	57.4 ± 5.0	62.4 ± 4.8	65.4 ± 4.6	60.4 ± 5.4
Lymphocytes (%)	34.2 ± 4.3	34.6 ± 4.0	32.0 ± 3.8	31.4 ± 3.3	33.2 ± 3.5
Cortisol (nmol/L)	513.1 ± 99.0	516.5 ± 109.6	661.8 ± 118.5	528.7 ± 93.8	511.2 ± 83.3
Total testosterone (nmol/L)	21.2 ± 4.1	18.9 ± 3.3	18.8 ± 3.0	21.5 ± 3.6	21.5 ± 4.0
T:C ratio	0.043 ± 0.010	0.039 ± 0.008	0.029 ± 0.005	0.040 ± 0.008	0.042 ± 0.008

The combined immune–metabolic response pattern across WBC, leukocyte differentials, lactate, urea, creatinine and irisin is summarised in Figure 6 and Figure 7.

**Table 4:** Neuromuscular–perceptual markers (mean ± SD).

Variable	T0 (Pre)	T1 (60 Min Pre-Match)	T2 (Post-Match)	T3 (24 h)	T4 (48 h)
Lactate (mmol/L)	1.4 ± 0.7	1.6 ± 0.5	10.9 ± 1.9	2.8 ± 0.6	1.8 ± 0.5
CMJ height (cm)	48.0 ± 2.1	47.6 ± 2.4	42.1 ± 2.1	41.8 ± 2.1	47.9 ± 2.5
20 m sprint (s)	2.93 ± 0.10	2.94 ± 0.11	3.08 ± 0.10	3.06 ± 0.11	2.94 ± 0.11
MVC (N)	2862.9 ± 298.2	2860.4 ± 288.1	2848.7 ± 280.8	2466.7 ± 263.0	2858.8 ± 282.5
DOMS (0–10)	0.5 ± 1.0	1.7 ± 1.2	6.5 ± 1.1	8.5 ± 1.0	4.5 ± 1.3

## Data Availability

The original contributions presented in this study are included in the article/Supplementary Materials. Further inquiries can be directed to the corresponding authors.

## Supplementary Materials

The following supporting information can be downloaded at: https://www.mdpi.com/article/10.3390/metabo16060370/s1, Table S1: Raw biochemical dataset across all time points (T0–T4); Table S2: Raw neuromuscular and perceptual data; Table S3: Physical baseline data of the players; Figure S1: Individual trajectories of muscle-damage and inflammatory markers; Figure S2: Expanded distribution (raincloud) plots for all biochemical markers; Figure S3: Extended biochemical heatmaps; Figure S4: Full correlation structure across biomarker systems; Figure S5: Extended biomarker–performance association plots; Figure S6: Full time-course panels for all biochemical and performance markers; Figure S7: Multi-dimensional fatigue fingerprints for individual players; Methods S1: Standardised testing protocols for neuromuscular measures; Methods S2: Full statistical analysis plan and code.
