# Supplementary material for: Acute Capillary Plasma Biomarker, Neuromuscular, and Perceptual Responses to Standardised Soccer Match Play in Elite Players: A Descriptive Study of Asynchronous Multi-Domain Recovery
Source: Metabolites. 2026 May 29;16(6):370. doi: 10.3390/metabo16060370 (PMC13303859; doi:10.3390/metabo16060370)
Supplement: Supplementary file 1 [file metabolites-16-00370-s001.zip › metabolites-4304101-supplementary.pdf]

Supplementary materials	Page
**Appendix 1. Table S1. Raw biochemical dataset across all time points (T0–T4).**	2-7
**Appendix 2. Table S2. Raw neuromuscular and perceptual data.**	8-10
**Appendix 3. Table S3. Physical baseline data of the players**	10-11
**Appendix 4. Figure S1. Individual trajectories of muscle-damage and inflammatory markers.**	12
**Appendix 5. Figure S2. Expanded distribution (raincloud) plots for all biochemical markers.**	13
**Appendix 6. Figure S3. Extended biochemical heatmaps.**	14-15
**Appendix 7. Figure S4. Full correlation structure across biomarker systems.**	16
**Appendix 8. Figure S5. Extended biomarker–performance association plots.**	17
**Appendix 9. Figure S6. Full time-course panels for all biochemical and performance markers.**	17
**Appendix 10. Figure S7. Multi-dimensional fatigue fingerprints for individual players.**	18
**Appendix 11. Methods S1. Standardised testing protocols for neuromuscular measures.**	19-20
**Appendix 12. Methods S2. Full statistical analysis plan and code.**	20-41

Appendix 1. Table S1. Raw biochemical dataset across all time points (T0–T4).

ID	Timepoint	CK_U_L	LDH_U_L	Myoglobin_ng_mL	IL6_pg_mL	TNF_alpha_pg_mL	hsCRP_mg_L	WBC_10e9_L	Neutrophil_pct	Lymphocyte_pct	Cortisol_nmol_L	TotalTestosterone_nmol_L	TC_ratio	Lactate_mmol_L	Urea_mmol_L	Creatinine_umol_L	Irisin_ng_mL
S01	T0	230.3	263.8	63.3	0.6	1.2	0.9	5.0	56.1	35.9	458.8	19.9	4.34%	1.2	6.3	81.5	3.5
S01	T1	275.9	258.1	68.7	1.0	0.9	1.3	5.6	55.6	38.5	523.1	18.9	3.62%	1.8	7.2	84.5	3.6
S01	T2	537.0	380.3	211.1	11.0	1.4	2.6	7.4	60.3	34.2	627.2	19.4	3.10%	10.2	7.9	92.7	10.2
S01	T3	603.9	407.3	283.8	5.2	1.3	7.0	4.8	65.2	34.4	515.9	21.8	4.22%	2.5	8.5	99.4	5.8
S01	T4	303.8	314.5	132.8	2.2	1.3	1.7	4.7	57.8	37.1	482.3	18.5	3.84%	2.0	7.7	92.9	4.5
S02	T0	202.6	212.5	49.9	0.7	1.2	1.1	7.6	51.2	32.9	662.9	18.7	2.83%	1.1	5.6	96.7	4.9
S02	T1	230.6	249.7	79.1	1.7	1.3	1.3	6.5	47.4	31.8	623.2	15.6	2.50%	1.1	6.1	96.8	4.7
S02	T2	449.1	307.2	254.9	12.5	1.5	2.3	9.0	55.4	30.8	792.6	15.4	1.94%	10.5	6.8	103.4	11.9
S02	T3	609.6	368.0	333.0	5.6	1.5	6.0	7.4	59.1	31.7	633.1	17.0	2.69%	2.5	7.4	98.2	7.3
S02	T4	331.2	261.5	171.8	3.2	1.4	1.7	7.3	51.2	33.0	629.9	17.6	2.79%	1.9	6.2	95.4	5.2
S03	T0	226.5	237.2	27.9	1.4	1.2	0.8	7.2	51.2	31.8	450.2	16.7	3.71%	0.7	5.3	110.0	4.1
S03	T1	267.7	249.6	41.9	1.7	1.5	0.8	7.6	50.7	31.0	460.0	16.5	3.60%	1.7	5.4	108.6	4.2
S03	T2	514.6	317.8	113.0	14.1	1.2	1.4	9.8	52.9	28.4	518.1	16.4	3.17%	10.5	6.0	106.2	10.8
S03	T3	733.2	402.0	160.3	6.2	1.5	4.7	8.3	58.4	31.5	464.8	18.1	3.90%	2.5	6.7	117.9	6.9
S03	T4	381.6	296.0	81.6	2.7	1.3	1.5	7.7	52.3	30.3	437.7	20.1	4.58%	1.4	5.5	106.2	4.2

S04	T0	257.7	227.3	40.0	2.0	1.5	0.6	6.5	53.7	31.7	508.0	23.5	4.62%	2.6	4.9	85.4	3.6
S04	T1	260.0	238.1	86.0	1.5	1.6	0.8	5.9	55.1	36.2	522.3	20.2	3.87%	2.1	5.3	92.4	4.1
S04	T2	543.7	315.9	198.1	15.0	1.7	1.7	9.3	59.8	34.2	691.9	21.6	3.11%	13.0	5.9	97.3	10.0
S04	T3	842.5	375.9	279.8	7.1	1.9	4.2	6.4	62.9	31.6	552.6	24.9	4.51%	3.6	6.6	105.8	6.5
S04	T4	406.3	246.5	112.2	4.1	1.6	0.8	6.8	56.9	33.5	560.1	23.9	4.26%	2.3	5.5	100.5	4.6
S05	T0	293.8	191.0	82.4	1.5	1.5	0.7	7.4	52.7	37.1	542.4	19.0	3.51%	1.8	6.9	99.4	4.3
S05	T1	374.6	199.5	84.5	1.8	1.5	0.2	6.1	55.2	37.6	453.1	18.4	4.07%	1.8	7.3	101.1	4.6
S05	T2	657.0	264.2	263.1	16.0	1.7	1.6	8.9	62.7	36.1	681.7	17.0	2.49%	10.8	8.2	110.3	11.5
S05	T3	863.9	325.8	374.0	9.2	1.7	3.7	7.3	65.5	36.5	538.9	19.4	3.60%	1.9	8.6	108.1	6.6
S05	T4	432.7	239.8	171.9	3.9	1.4	1.2	6.9	61.2	41.0	561.9	19.3	3.44%	1.8	7.5	96.0	4.4
S06	T0	438.8	176.0	16.1	1.6	1.4	1.0	7.0	55.0	32.4	579.1	22.5	3.88%	2.0	5.9	105.0	4.9
S06	T1	466.9	170.9	27.4	2.1	1.4	0.5	7.3	58.8	34.7	575.7	18.7	3.25%	2.4	6.1	108.1	4.2
S06	T2	897.0	223.0	106.5	14.8	1.7	1.9	10.1	58.9	29.1	735.5	18.8	2.55%	13.3	7.1	111.7	11.5
S06	T3	1091.9	267.5	147.0	8.1	1.8	4.6	7.1	61.8	25.9	572.3	20.6	3.60%	4.2	7.5	120.9	6.3
S06	T4	652.4	171.1	65.8	3.7	1.6	1.1	6.7	61.9	28.9	560.1	20.0	3.58%	1.7	6.4	115.3	5.4
S07	T0	310.3	226.5	55.7	0.7	0.9	1.0	6.3	60.8	34.1	479.6	22.6	4.71%	1.1	6.0	112.9	5.4
S07	T1	372.8	238.0	66.3	1.2	0.9	1.2	5.7	61.3	34.7	478.6	19.1	3.99%	2.3	6.1	111.8	5.5
S07	T2	829.7	314.4	192.3	14.6	0.9	1.8	8.2	63.2	30.1	617.7	20.4	3.30%	11.9	7.0	125.7	13.1
S07	T3	1015.6	387.1	283.7	7.5	1.3	4.7	6.4	63.3	32.6	475.3	22.7	4.78%	2.8	7.5	127.6	8.3

S07	T4	591.8	287.5	132.5	3.1	0.9	1.6	5.9	65.0	30.8	434.7	24.2	5.58%	1.9	6.6	114.0	5.6
S08	T0	108.5	172.8	25.9	0.4	1.1	0.2	5.7	65.6	33.3	532.3	16.9	3.17%	1.8	6.0	75.6	4.4
S08	T1	185.3	144.7	35.0	1.1	1.1	0.6	6.5	65.1	35.4	560.5	17.1	3.05%	1.5	5.9	86.4	4.7
S08	T2	330.2	226.6	137.8	7.5	1.3	0.6	8.3	69.5	31.9	683.7	14.5	2.12%	10.4	7.0	91.2	10.9
S08	T3	553.8	282.5	181.5	4.1	1.5	1.5	6.1	77.1	32.4	515.7	17.4	3.37%	2.7	7.6	91.7	7.7
S08	T4	273.4	182.9	77.6	1.9	1.1	0.4	6.5	66.0	34.3	471.4	20.0	4.24%	1.8	6.5	82.2	5.3
S09	T0	287.4	210.9	38.1	2.0	1.2	0.4	7.9	62.0	33.2	470.3	18.8	4.00%	1.7	5.8	84.9	5.1
S09	T1	311.7	213.5	39.8	2.2	1.2	0.6	7.5	59.6	35.2	482.9	17.2	3.56%	1.1	5.9	90.9	5.3
S09	T2	606.1	276.9	145.3	19.7	1.5	1.5	9.0	65.3	31.4	596.5	16.9	2.83%	6.7	7.1	82.0	13.6
S09	T3	814.7	334.7	210.1	10.4	1.5	3.8	7.7	70.2	32.3	474.4	15.7	3.31%	1.9	7.6	96.7	8.5
S09	T4	415.3	252.5	75.9	4.4	1.3	1.2	8.5	66.4	29.6	459.2	16.9	3.68%	1.1	6.5	90.7	6.2
S10	T0	251.5	258.6	59.5	0.9	0.9	0.0	7.0	46.6	32.9	453.2	26.7	5.88%	1.2	4.9	92.3	4.5
S10	T1	325.9	260.8	67.4	1.8	0.8	0.4	7.2	51.8	36.2	491.1	22.0	4.48%	1.1	5.3	96.7	4.7
S10	T2	674.8	344.0	215.9	14.3	0.9	1.0	9.0	52.4	30.0	628.7	21.9	3.49%	7.7	5.9	100.9	11.5
S10	T3	927.3	402.3	298.8	6.9	0.8	3.2	8.1	55.6	32.3	496.1	26.3	5.31%	2.4	6.5	106.3	7.7
S10	T4	528.7	317.3	126.8	2.3	0.9	0.5	7.4	54.2	34.0	484.7	26.3	5.44%	1.4	5.1	96.3	5.5
S11	T0	332.1	294.0	39.5	1.0	0.9	0.1	7.4	58.6	42.0	485.9	21.5	4.42%	2.4	6.4	96.6	3.9
S11	T1	404.5	298.6	60.5	1.0	1.1	0.7	8.2	61.3	36.3	524.2	19.2	3.66%	1.4	6.5	100.1	4.6
S11	T2	790.2	409.6	170.2	9.5	1.1	1.2	11.1	68.3	36.9	638.1	17.7	2.77%	14.0	7.6	106.9	11.2

S11	T3	981.5	474.3	251.0	5.3	1.6	1.8	8.1	72.1	33.4	517.3	19.3	3.74%	3.3	8.2	112.4	6.6
S11	T4	500.9	356.7	90.8	1.8	1.3	0.3	7.2	67.1	34.7	510.7	21.7	4.25%	3.2	6.9	105.8	5.5
S12	T0	281.1	265.9	36.5	1.8	1.4	0.5	6.5	57.3	32.5	562.2	18.8	3.34%	0.7	5.8	101.0	3.9
S12	T1	184.0	270.4	68.7	2.5	1.6	0.6	6.3	57.6	33.4	504.8	17.2	3.41%	0.8	6.0	111.8	4.8
S12	T2	440.1	375.7	200.1	19.9	1.7	1.5	8.3	60.6	30.6	715.2	19.3	2.70%	8.9	7.0	117.4	11.0
S12	T3	590.7	449.8	270.0	10.2	1.9	4.1	6.4	64.4	28.7	540.5	20.2	3.74%	3.0	7.3	120.3	6.6
S12	T4	342.1	313.4	116.1	4.3	1.6	1.1	7.1	63.1	33.4	481.2	19.0	3.94%	1.9	6.4	110.5	4.9
S13	T0	228.5	204.3	42.6	1.2	1.2	0.8	8.8	54.4	39.9	543.9	24.3	4.46%	1.8	5.8	106.1	4.3
S13	T1	227.4	206.5	57.1	0.9	1.1	1.1	8.1	55.9	37.2	520.7	21.6	4.15%	1.4	5.8	108.9	4.6
S13	T2	510.5	299.8	182.3	10.0	1.2	2.0	10.8	64.8	37.6	667.8	21.7	3.24%	10.3	6.7	119.6	11.2
S13	T3	688.6	313.5	267.4	5.4	1.3	5.6	8.0	66.9	33.7	505.0	24.4	4.83%	2.7	7.4	122.1	7.8
S13	T4	384.9	242.6	122.3	2.6	1.3	1.7	8.6	60.8	37.2	543.8	22.3	4.09%	2.0	6.5	115.0	5.1
S14	T0	200.0	260.3	48.8	1.2	0.9	1.0	5.5	60.8	34.5	555.9	24.0	4.32%	2.5	5.4	105.2	3.8
S14	T1	342.3	256.7	70.7	1.9	1.0	1.1	5.9	62.8	36.3	564.2	20.8	3.69%	2.1	5.6	105.0	4.2
S14	T2	513.8	356.4	208.9	16.7	1.5	2.1	8.1	68.1	33.1	755.9	20.9	2.77%	14.1	6.5	104.7	9.6
S14	T3	641.9	402.5	300.3	9.3	1.3	6.0	5.3	68.2	33.8	626.4	25.3	4.04%	4.4	7.0	115.8	5.2
S14	T4	325.3	286.7	124.9	3.3	1.2	1.7	5.6	60.6	32.0	586.5	23.8	4.06%	2.6	6.0	105.3	4.7
S15	T0	349.6	165.7	24.9	1.0	1.3	1.3	8.2	58.9	35.1	536.0	20.4	3.80%	1.6	5.6	99.8	3.1
S15	T1	284.1	178.8	35.0	1.3	1.4	1.1	6.6	59.5	33.1	569.5	17.4	3.05%	1.6	5.6	106.8	3.5

S15	T2	727.2	266.1	107.1	13.1	1.5	1.9	10.1	66.9	32.7	699.8	17.4	2.49%	11.8	6.7	109.5	7.7
S15	T3	850.4	297.0	150.5	6.6	1.8	5.6	7.9	68.6	31.6	593.0	21.7	3.65%	3.0	7.3	108.3	4.7
S15	T4	533.3	230.0	65.9	2.5	1.5	1.5	8.1	57.8	30.2	533.0	19.6	3.68%	2.5	5.9	101.9	3.8
S16	T0	320.1	230.1	44.2	1.4	1.0	0.6	7.4	52.3	28.8	497.0	15.7	3.17%	1.2	5.9	118.9	3.8
S16	T1	406.7	240.1	61.0	0.0	0.9	0.6	7.1	52.8	32.1	528.0	15.2	2.87%	2.1	5.9	122.0	3.8
S16	T2	605.7	340.7	185.6	3.3	0.8	1.6	9.6	59.1	26.8	655.6	17.3	2.64%	11.5	6.7	123.2	9.7
S16	T3	947.8	375.4	257.2	1.4	1.2	3.7	7.7	57.1	29.0	539.1	17.5	3.26%	2.6	7.1	139.0	5.6
S16	T4	554.9	280.8	117.5	-0.1	1.1	0.5	7.4	51.2	31.2	521.6	17.5	3.35%	1.5	6.4	121.7	4.9
S17	T0	249.3	216.5	67.4	1.8	1.1	0.4	6.8	53.8	36.5	405.1	21.6	5.33%	0.7	6.0	85.2	4.0
S17	T1	163.0	237.3	76.3	2.6	1.1	0.9	7.2	55.1	33.8	421.2	16.7	3.97%	1.1	6.2	90.5	4.3
S17	T2	508.7	296.4	241.3	17.4	1.2	2.1	8.1	63.4	30.3	533.9	19.1	3.59%	9.8	7.1	95.9	10.8
S17	T3	708.3	346.8	347.8	9.1	1.3	4.7	7.0	61.8	31.6	427.0	21.6	5.05%	2.4	7.5	94.2	7.0
S17	T4	458.1	274.2	145.4	4.3	1.2	1.3	6.6	62.7	35.3	372.5	21.7	5.82%	1.6	6.5	84.5	5.3
S18	T0	264.0	224.1	42.9	1.6	1.2	0.7	6.3	57.2	28.9	526.3	16.5	3.13%	1.8	6.9	85.8	5.1
S18	T1	339.4	249.3	71.8	1.8	1.1	0.3	6.2	58.5	33.6	534.6	15.8	2.95%	1.5	7.1	87.9	5.0
S18	T2	602.0	307.3	210.1	16.5	1.3	0.9	8.5	61.0	30.0	716.0	12.6	1.76%	11.6	8.3	95.0	11.6
S18	T3	727.5	379.6	280.3	8.5	1.4	2.6	6.2	70.0	26.4	517.7	19.3	3.74%	3.5	9.0	93.8	7.5
S18	T4	392.6	274.8	117.6	4.1	1.1	0.5	4.7	62.1	31.6	541.1	19.2	3.56%	1.2	7.7	83.9	5.2
S19	T0	346.4	248.3	30.0	1.1	1.1	1.0	7.4	61.1	30.4	539.9	23.7	4.39%	0.8	6.3	118.1	5.3

S19	T1	343.6	242.5	28.6	2.3	1.2	0.9	7.1	63.7	31.0	527.3	21.0	3.98%	0.9	6.6	111.2	5.3
S19	T2	709.3	377.4	137.9	16.0	1.2	2.6	10.1	69.4	30.3	736.2	20.0	2.72%	9.0	7.5	123.6	13.0
S19	T3	901.0	413.1	176.8	7.1	1.4	6.5	8.0	65.5	25.3	572.8	23.5	4.11%	2.3	7.9	132.9	8.3
S19	T4	540.0	307.0	74.4	3.5	1.2	1.7	7.0	68.2	30.9	624.6	24.0	3.85%	1.4	6.7	114.9	5.6
S20	T0	271.8	248.7	92.4	1.8	1.1	1.3	7.2	55.1	37.3	503.3	20.0	3.97%	1.3	5.4	78.9	4.6
S20	T1	421.5	256.2	100.9	2.2	1.3	1.2	7.9	57.7	34.6	473.9	18.8	3.96%	1.7	5.6	79.8	4.0
S20	T2	797.9	371.1	300.1	15.4	1.3	2.1	10.2	59.9	33.4	614.0	19.2	3.12%	10.6	6.7	83.9	11.7
S20	T3	1046.1	426.5	438.5	8.0	1.3	5.2	8.0	66.3	34.6	479.6	21.4	4.47%	2.6	6.9	87.9	6.5
S20	T4	526.8	319.4	177.1	3.6	1.4	1.4	8.4	59.3	34.2	451.4	21.2	4.69%	1.6	5.9	87.0	4.5
S21	T0	171.6	249.5	31.3	0.8	1.1	0.1	6.2	60.3	38.3	527.8	29.2	5.52%	0.5	5.8	122.2	4.1
S21	T1	172.7	235.1	35.6	2.1	1.0	-0.1	5.9	57.7	34.0	546.7	26.8	4.89%	1.2	6.2	121.0	3.8
S21	T2	423.7	356.8	130.7	14.1	1.5	0.3	8.8	67.0	35.1	594.0	25.6	4.30%	13.3	7.3	127.1	10.1
S21	T3	478.7	420.9	158.6	7.7	1.5	0.9	7.8	68.9	31.2	568.8	29.6	5.20%	2.8	7.7	139.2	5.7
S21	T4	247.9	298.3	78.9	3.1	1.2	0.7	7.2	63.1	35.1	527.0	30.3	5.76%	1.7	6.8	131.9	4.5
S22	T0	273.7	250.7	41.7	1.7	1.2	1.2	5.2	58.2	33.2	468.5	25.5	5.44%	0.2	7.1	80.1	3.7
S22	T1	310.7	257.3	49.7	2.1	1.2	0.9	5.8	60.2	34.0	476.6	21.3	4.47%	1.1	7.1	88.4	5.1
S22	T2	648.2	355.5	114.3	17.5	1.4	2.0	8.1	64.6	31.7	659.0	20.1	3.04%	10.2	8.4	92.7	10.5
S22	T3	803.4	416.1	151.9	8.6	1.5	4.8	6.2	69.6	31.0	505.7	24.8	4.90%	2.6	9.0	104.7	7.5
S22	T4	453.6	310.4	65.2	3.6	1.3	1.5	6.6	60.4	33.0	470.9	25.2	5.36%	2.0	7.8	91.5	4.9

Appendix 2. Table S2. Raw neuromuscular and perceptual data.

Subject_ID	Timepoint	CMJ_cm	Sprint20m_s	MVC_N	DOMS
S01	T0	46.9	3.1	3337.5	0.7
S01	T1	47.8	3.1	3335.9	1.9
S01	T2	40.9	3.2	3366.3	6.7
S01	T3	42.7	3.2	2921.9	8.7
S01	T4	46.0	3.0	3344.9	4.7
S02	T0	51.4	2.9	3052.9	0.5
S02	T1	50.8	2.9	3091.6	1.7
S02	T2	45.1	3.1	3106.7	6.5
S02	T3	44.8	3.1	2623.9	8.5
S02	T4	52.7	3.0	3068.4	4.5
S03	T0	46.4	2.9	2846.6	0.7
S03	T1	45.8	2.9	2874.2	1.9
S03	T2	40.0	3.1	2745.1	6.7
S03	T3	40.7	3.1	2434.7	8.7
S03	T4	47.7	3.0	2724.3	4.7
S04	T0	50.1	2.9	2551.4	0.4
S04	T1	48.1	2.9	2473.5	1.6
S04	T2	43.7	3.1	2514.3	6.4
S04	T3	41.5	3.1	2163.6	8.4
S04	T4	48.1	3.0	2566.1	4.4
S05	T0	44.6	2.9	2816.9	0.3
S05	T1	46.3	2.9	2702.5	1.5
S05	T2	37.5	3.0	2676.5	6.3
S05	T3	38.1	3.0	2411.4	8.3
S05	T4	43.2	2.9	2747.9	4.3
S06	T0	46.9	2.9	2551.4	0.4
S06	T1	45.2	3.0	2545.9	1.6
S06	T2	40.7	3.1	2634.2	6.4
S06	T3	41.8	3.1	2199.9	8.4
S06	T4	45.4	3.0	2578.2	4.4
S07	T0	51.5	2.9	2532.7	0.7
S07	T1	53.1	2.9	2632.5	1.9
S07	T2	44.7	3.1	2603.0	6.7
S07	T3	45.1	3.1	2231.5	8.7
S07	T4	52.6	2.9	2524.2	4.7
S08	T0	48.5	2.9	2860.5	-0.1
S08	T1	49.8	2.9	2849.7	1.1
S08	T2	42.7	3.1	2839.7	5.9

S08	T3	43.9	3.1	2385.0	7.9
S08	T4	49.8	2.9	2918.9	3.9
S09	T0	47.0	2.8	3154.8	0.4
S09	T1	47.2	2.9	3184.9	1.6
S09	T2	40.3	2.9	3204.6	6.4
S09	T3	39.4	2.9	2723.4	8.4
S09	T4	48.7	2.9	3160.3	4.4
S10	T0	49.5	2.9	2956.2	0.1
S10	T1	49.3	2.9	3026.3	1.3
S10	T2	44.0	3.0	3010.3	6.1
S10	T3	43.5	3.0	2537.9	8.1
S10	T4	49.3	2.9	2977.8	4.1
S11	T0	48.0	3.0	2772.0	0.8
S11	T1	44.2	3.0	2790.6	2.0
S11	T2	42.2	3.1	2756.6	6.8
S11	T3	40.2	3.1	2436.5	8.8
S11	T4	47.6	3.0	2728.2	4.8
S12	T0	50.5	2.9	2729.8	0.4
S12	T1	49.7	2.9	2614.6	1.6
S12	T2	42.2	3.0	2694.0	6.4
S12	T3	44.1	3.0	2294.0	8.4
S12	T4	50.4	2.9	2624.7	4.4
S13	T0	48.8	2.9	2820.8	0.9
S13	T1	47.3	2.9	2792.8	2.1
S13	T2	43.0	3.1	2829.3	6.9
S13	T3	41.9	3.0	2464.0	8.9
S13	T4	48.3	2.9	2858.8	4.9
S14	T0	46.4	3.0	3012.3	0.9
S14	T1	46.3	3.0	3062.0	2.1
S14	T2	42.7	3.1	3042.0	6.9
S14	T3	40.3	3.1	2618.0	8.9
S14	T4	45.9	3.0	3048.0	4.9
S15	T0	43.4	3.0	3054.9	0.1
S15	T1	43.6	3.0	2903.9	1.3
S15	T2	40.2	3.2	2990.0	6.1
S15	T3	38.4	3.1	2590.3	8.1
S15	T4	44.6	3.0	2997.1	4.1
S16	T0	46.3	3.0	2738.4	0.8
S16	T1	43.9	3.0	2760.5	2.0
S16	T2	38.7	3.2	2700.4	6.8
S16	T3	38.0	3.1	2353.7	8.8

S16	T4	45.0	3.0	2767.6	4.8
S17	T0	46.3	2.9	2599.5	0.5
S17	T1	45.6	2.9	2695.9	1.7
S17	T2	40.4	3.1	2591.9	6.5
S17	T3	40.4	3.0	2259.6	8.5
S17	T4	46.4	2.9	2600.2	4.5
S18	T0	47.6	3.0	2919.4	0.5
S18	T1	47.8	3.0	2818.4	1.7
S18	T2	43.3	3.2	2862.5	6.5
S18	T3	41.9	3.1	2475.4	8.5
S18	T4	47.5	3.0	2823.6	4.5
S19	T0	47.8	2.9	2889.5	0.5
S19	T1	48.9	2.9	2924.9	1.7
S19	T2	42.2	3.0	2932.0	6.5
S19	T3	42.7	3.0	2550.4	8.5
S19	T4	51.3	2.9	2987.0	4.5
S20	T0	50.5	3.0	2835.8	0.5
S20	T1	49.5	3.0	2980.5	1.7
S20	T2	45.8	3.1	2814.5	6.5
S20	T3	44.5	3.1	2528.3	8.5
S20	T4	49.7	3.0	2896.8	4.5
S21	T0	48.1	2.8	2940.5	0.4
S21	T1	48.1	2.9	2985.3	1.6
S21	T2	44.4	3.0	2892.4	6.4
S21	T3	43.4	3.0	2580.3	8.4
S21	T4	48.2	2.8	3015.6	4.4
S22	T0	48.6	2.8	3009.9	0.4
S22	T1	48.3	2.9	2882.9	1.6
S22	T2	40.7	3.0	2864.6	6.4
S22	T3	41.8	3.0	2484.6	8.4
S22	T4	46.4	2.9	2935.7	4.4

Appendix 3. Table S3. Physical baseline data of the players

Subject_ID	Age_years	Height_cm	BodyMass_kg	BMI_kg_m2	BodyFat_pct	TrainingAge_years	VO2max_ml_kg_min	HRmax_bpm
S01	24.8	180.3	75.2	23.1	11	10.1	60.9	202
S02	24.2	180.1	78.1	24.1	10.8	10.5	57.1	188
S03	24.8	180.4	79.9	24.6	12.5	9.4	56.8	199
S04	24.5	184.5	79	23.2	12.1	11.4	59	197
S05	22.2	180.4	77.9	23.9	11	11.2	65	201
S06	22.1	173.5	71.6	23.8	8.7	14.6	55.3	198
S07	22.6	182.7	81.6	24.4	12.1	11.2	57.5	195
S08	24.5	179.7	71.6	22.2	11.2	8.8	63.8	193
S09	23	179.9	78.3	24.2	12.7	8.9	57.4	190
S10	28.2	181.6	71.5	21.7	10.4	8.3	57.7	196
S11	21.9	182.2	67.6	20.4	14	11.9	59.3	194
S12	21.3	186.8	79.5	22.8	9.9	8.9	54.7	203
S13	25	184.3	76.1	22.4	13.4	9.9	59.8	193
S14	26.3	181.6	81	24.6	10.7	11.5	54.8	196
S15	24.5	177.3	76.4	24.3	9.6	8.6	62.1	189
S16	25.2	175.9	68.2	22	8.5	9.4	59	193
S17	26.4	182.2	81.4	24.5	10.2	6.3	67	196
S18	23.3	187.6	78.5	22.3	13.1	7.8	60	198
S19	20.7	181.2	76.8	23.4	12	8.9	63.8	201
S20	22.4	180.5	75.4	23.1	11.7	10.8	54.4	195
S21	25.4	183.7	79.8	23.6	6.2	12.4	58.6	191
S22	20.6	173.7	73.9	24.5	11.8	10	60.1	197

Appendix 4. Figure S1. Individual trajectories of muscle-damage and inflammatory markers.

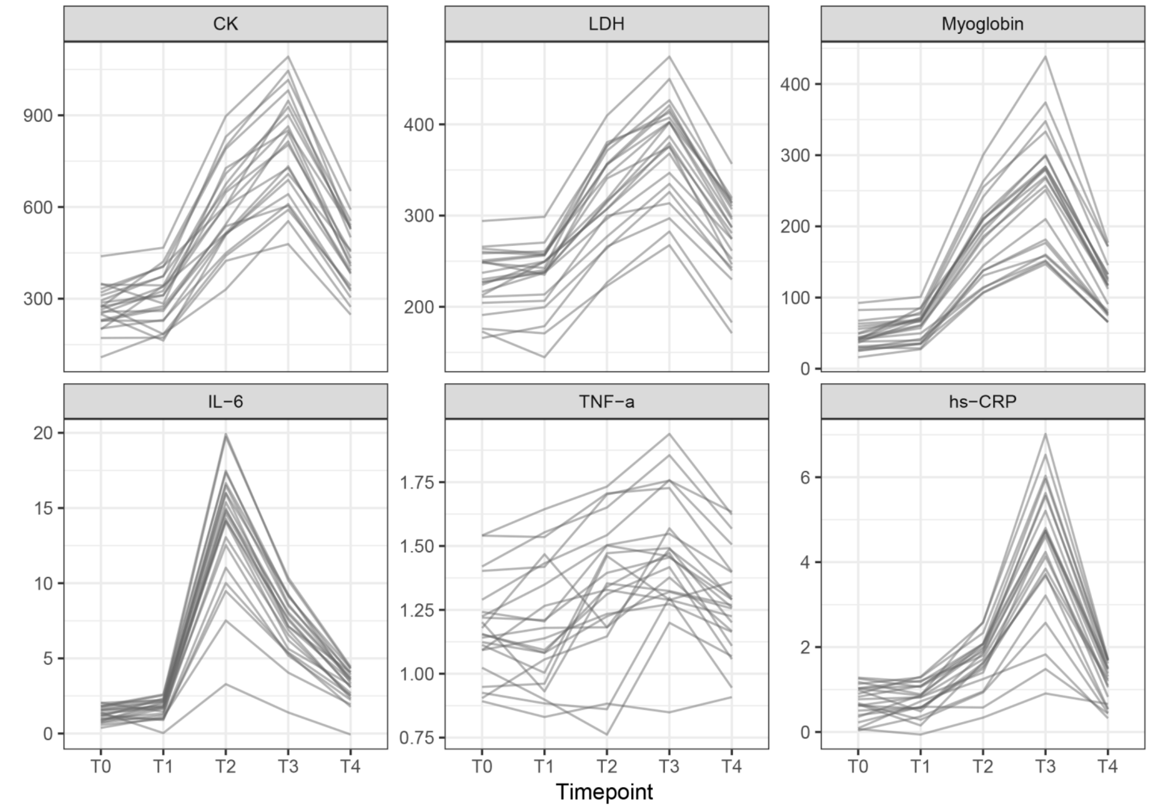

Appendix 5. Figure S2. Expanded distribution (raincloud) plots for all biochemical markers.

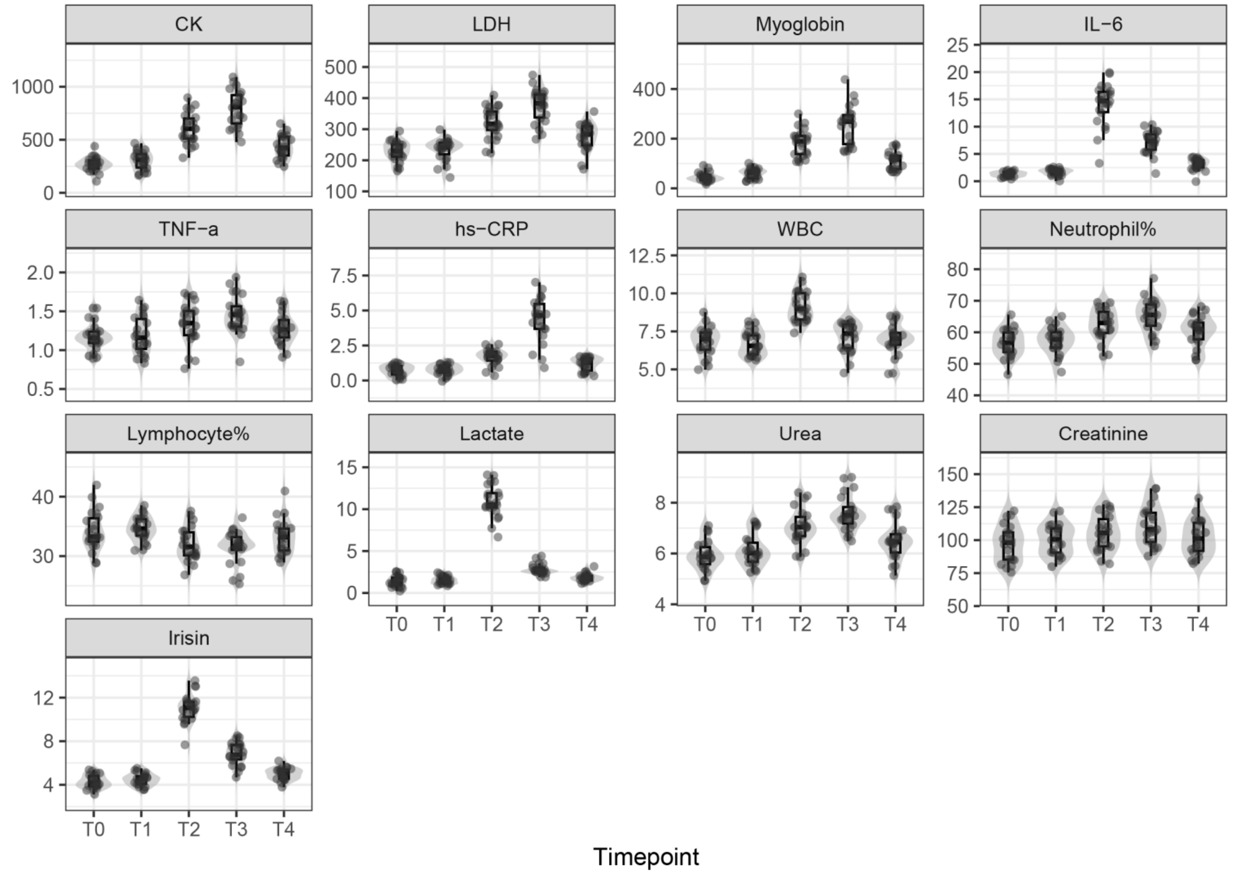

Appendix 6. Figure S3. Extended biochemical heatmaps.

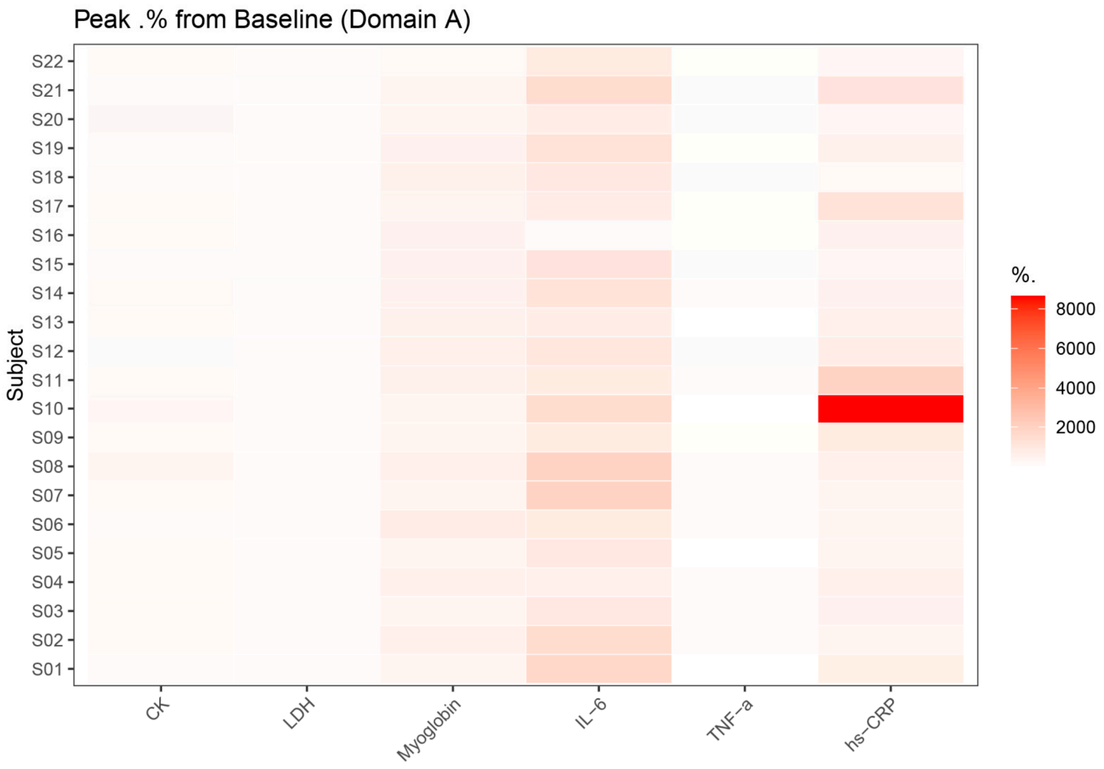

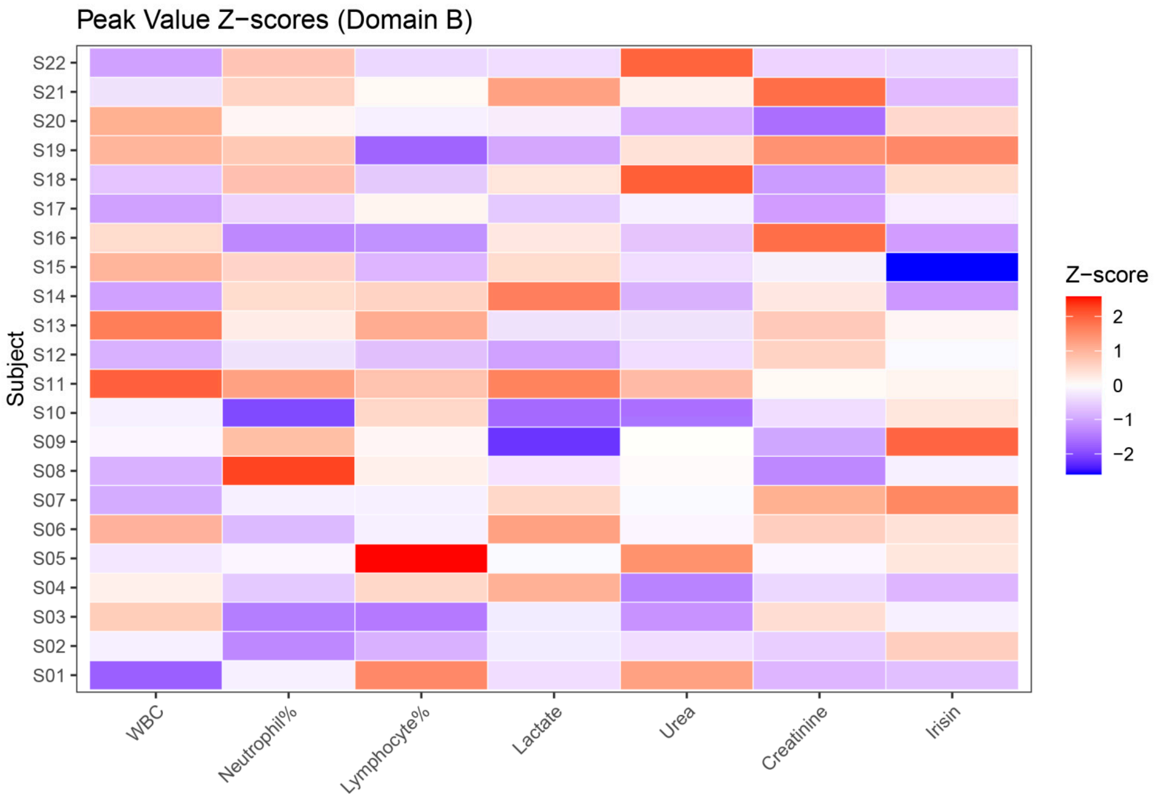

Appendix 7. Figure S4. Full correlation structure across biomarker systems.

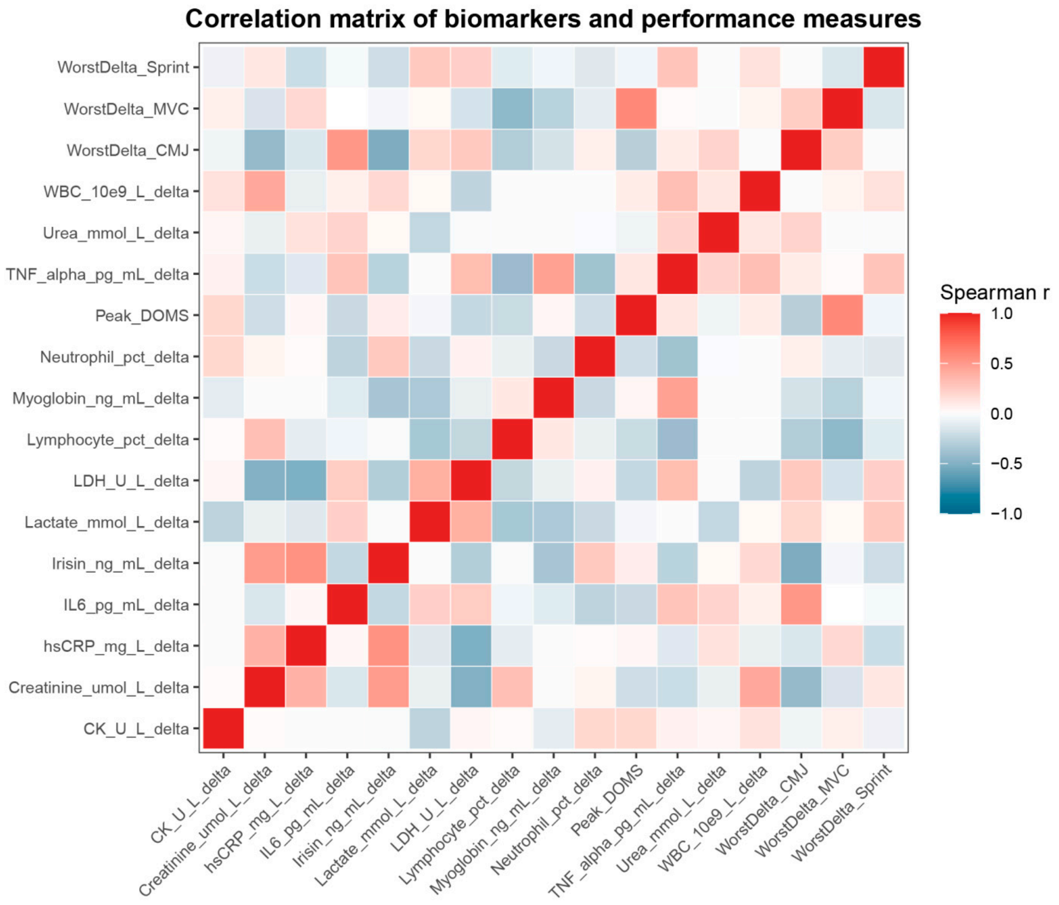

Appendix 8. Figure S5. Extended biomarker–performance association plots.

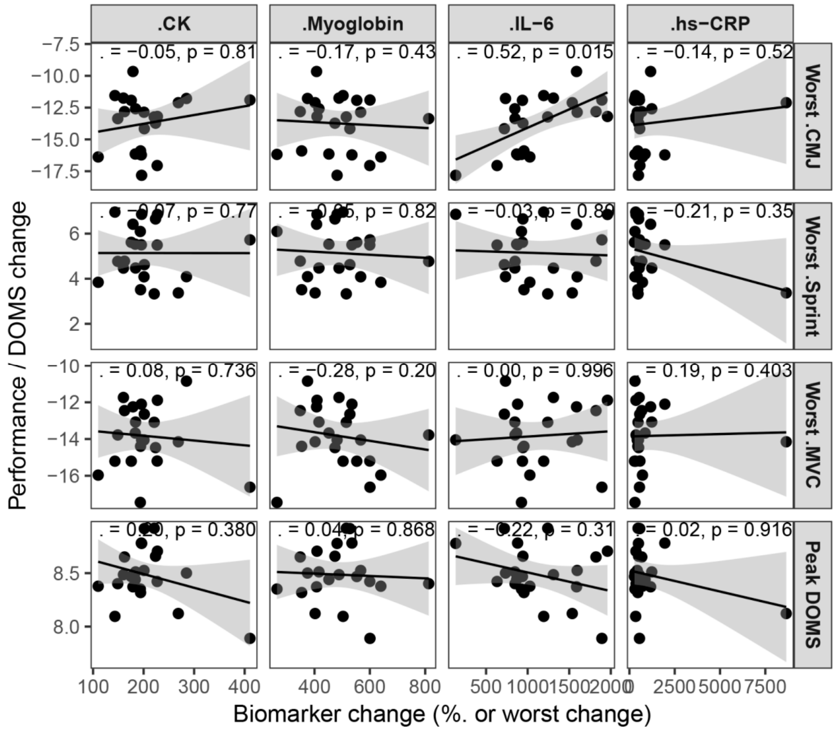

Appendix 9. Figure S6. Full time-course panels for all biochemical and performance markers.

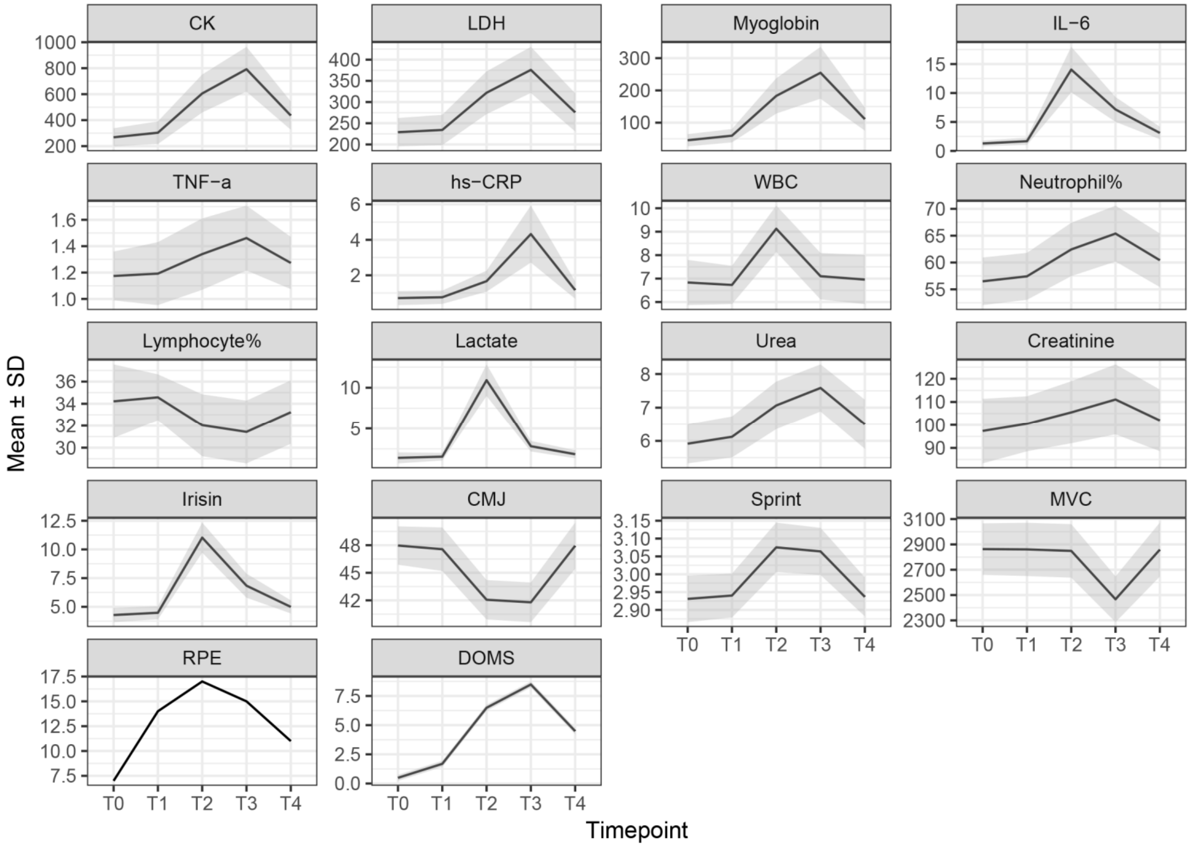

Appendix 10. Figure S7. Multi-dimensional fatigue fingerprints for individual players.

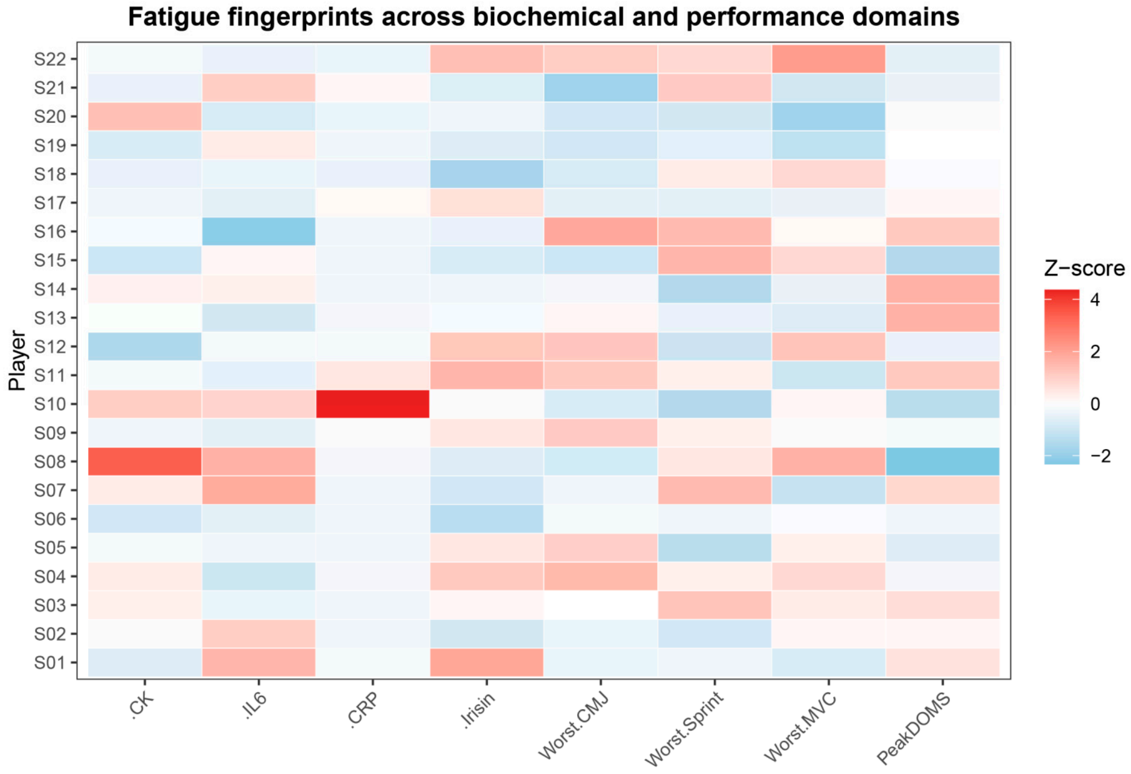

Appendix 11. Methods S1. Standardised testing protocols for neuromuscular measures.

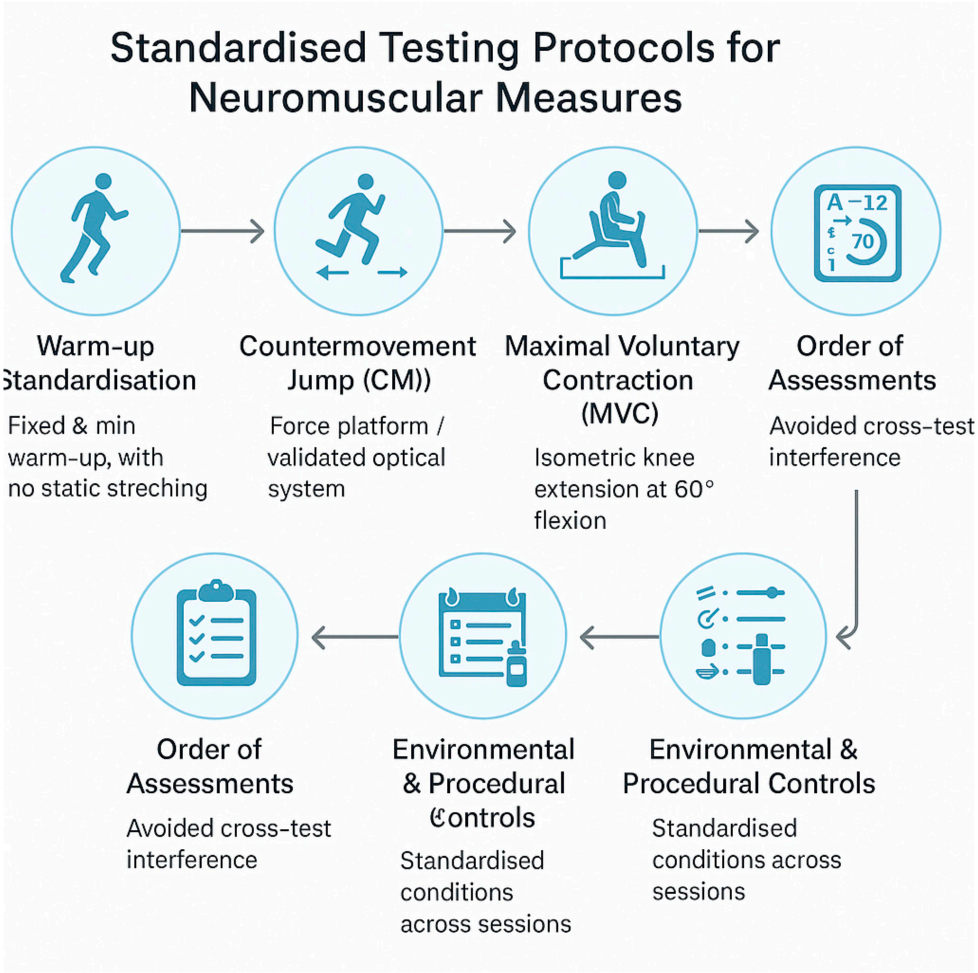

Neuromuscular assessments were conducted under strictly standardised conditions across all time points (T0–T4) to minimise technical, environmental, and biological variability. All evaluations took place in the same controlled indoor environment, on identical testing surfaces and at matched times of day relative to the competitive fixture. Players were already familiarised with all procedures as part of routine performance monitoring.

Prior to each testing session, athletes completed a fixed 8-min warm-up consisting of light jogging, dynamic mobility drills targeting major lower-limb joints, movement-specific preparation (low-intensity jumps, skips and accelerations), and a final test-specific activation phase aligned to the forthcoming assessment (submaximal CMJs, accelerations or isometric contractions). No static stretching or potentiation exercises were permitted to avoid altering neuromuscular readiness.

Countermovement jump performance was measured on a force platform or validated optical system, with athletes maintaining hands on hips and performing a self-selected countermovement without exceeding approximately 90° of knee flexion. Three maximal attempts were executed with 45 s passive recovery; trials involving arm swing or loss of balance were repeated. The best valid jump was retained, and force-platform sampling frequency remained ≥1000 Hz throughout, with all assessments conducted by the same technician.

Linear sprint performance was evaluated over 20 m using dual-beam electronic timing gates positioned at 0 m and 20 m. Athletes commenced from a standing start with the dominant foot placed 0.5 m behind the first gate, completing two maximal sprints separated by 2 min rest. The fastest trial was analysed, and attempts involving premature body movement (“rocking”) were invalidated. All sprint tests were performed on the same standardised artificial-turf lane, with footwear held constant.

Maximal voluntary contraction (MVC) of the knee extensors was assessed using an isometric dynamometer or calibrated custom rig. Players were seated with the hip positioned at 90° and the knee fixed at 60° flexion (0° = full extension), with pelvic and upper-body stabilisation to prevent compensatory motion. Following two submaximal familiarisation contractions (50–70%), participants performed three maximal efforts lasting 3–4 s each, separated by 90 s recovery. The highest instantaneous force was used for analysis. Joint angle was verified with a digital goniometer for every participant, and a trial-to-trial coefficient of variation <5% was required for data acceptance.

Perceptual responses were assessed via standardised ratings of perceived exertion (RPE, 6–20 scale) and delayed-onset muscle soreness (DOMS, 0–10 numerical rating scale). RPE was obtained immediately following each match half (T1, T2) and again at T3–T4 for daily activities, with players isolated from teammates to avoid anchoring effects. DOMS was recorded following three controlled functional tasks (walking, bodyweight squat, step-down), and athletes were instructed to report global lower-limb soreness rather than a single muscle group. All perceptual assessments were performed by the same assessor.

To avoid cross-test interference, the order of assessments remained fixed at all time points: (1) perceptual measures (RPE, DOMS), (2) capillary blood sampling, (3) CMJ, (4) 20-m sprint, and (5) MVC. This sequence ensured that metabolically demanding or fatiguing tasks did not influence biochemical sampling or earlier neuromuscular outcomes.

Environmental and procedural controls were rigorously maintained. Testing was always scheduled within ±30 min of each corresponding time point; players abstained from caffeine and alcohol for 24 h prior to assessments; dietary intake was standardised using the club's pre-training nutrition template; and hydration status was screened visually using a urine colour chart prior to T0 and T1, with rehydration mandated if necessary. All evaluations were administered by the same pair of trained assessors to minimise between-tester variability.

Appendix 12. Methods S2. Full statistical analysis plan and code.

```
############################################################

## Methods S2. Full statistical analysis plan and code

############################################################

## Load required packages

library(ggplot2)

library(dplyr)

library(tidyr)

library(reshape2)

library(RColorBrewer)

library(tibble)

############################################################

## 1. Data import and basic structure

############################################################

setwd("C:/Users/LENOVO/Desktop")

df <- read.csv("elitesoccer.csv", header = TRUE, stringsAsFactors = FALSE)

df$Subject_ID <- as.factor(df$Subject_ID)

df$Timepoint  <- factor(df$Timepoint, levels = c("T0","T1","T2","T3","T4"))

## Colors for biomarker plots

bg_col   <- "grey90"

line_col <- "black"

err_col  <- "black"

############################################################

## 2. Example: CK mean, SD and bar + line plot with p-values
```

```
##########################################################
## Extract CK and compute mean ± SD across time points
ck <- df[, c("Timepoint","CK_U_L")]
ck_mean <- tapply(ck$CK_U_L, ck$Timepoint, mean)
ck_sd   <- tapply(ck$CK_U_L, ck$Timepoint, sd)
ck_mean
ck_sd
## Plot CK using base R (bar + line + SD + significance stars)
par(xaxs = "i", yaxs = "i")
bar_centers <- barplot(
ck_mean,
col    = bg_col,
border = NA,
ylim   = c(0, max(ck_mean + ck_sd) * 1.6),
xlab   = "",
ylab   = "CK (U/L)",
main   = "Creatine Kinase Post-Match Response",
cex.main = 1.6,
cex.lab  = 1.4,
axes   = FALSE,
xaxt   = "n"
)
axis(2, cex.axis = 1.2)
axis(1, at = bar_centers, labels = levels(df$Timepoint), cex.axis = 1.3)
box()
lines(bar_centers, ck_mean, type = "b",
pch = 16, col = line_col, lwd = 2.5)
arrows(bar_centers, ck_mean - ck_sd,
bar_centers, ck_mean + ck_sd,
angle = 90, code = 3, length = 0.1,
lwd = 2.2, col = err_col)


## Significance stars for T1–T4 vs T0 (example p-values)
p_vals <- c(0.01896, 4.768e-07, 4.768e-07, 4.768e-07)


p_stars <- ifelse(
```

```
p_vals < 0.001, "***",
ifelse(
p_vals < 0.01, "**",
ifelse(p_vals < 0.05, "*", "ns")
)
)

target_bars <- bar_centers[2:5]

star_y <- ck_mean[2:5] + ck_sd[2:5] + max(ck_sd) * 0.2

for (i in 1:4) {
text(target_bars[i], star_y[i], p_stars[i], cex = 1.8)
}

############################################################
## 3. Generic function for time-course biomarker plots
##    (Friedman + Wilcoxon + bar + line + SD + stars)
############################################################

plot_biomarker <- function(df, var_name, y_label, main_title) {

## Extract data and compute mean ± SD
dat <- df[, c("Subject_ID", "Timepoint", var_name)]
colnames(dat)[3] <- "value"

mean_vals <- tapply(dat$value, dat$Timepoint, mean)
sd_vals   <- tapply(dat$value, dat$Timepoint, sd)

## Reshape to wide format: rows = subjects, cols = time points
wide <- reshape(
dat,
idvar     = "Subject_ID",
timevar   = "Timepoint",
direction = "wide"
)
```

```
tp_levels <- levels(df$Timepoint)
cols      <- paste0("value.", tp_levels)
wide      <- wide[complete.cases(wide[, cols]), ]
mat       <- as.matrix(wide[, cols])
colnames(mat) <- tp_levels
## Friedman test for overall time effect
cat("\n==============================\n")
cat("Indicator:", var_name, "\n")
print(friedman.test(mat))

## Wilcoxon tests: T0 vs T1–T4
baseline <- mat[, "T0"]
p_vals   <- numeric(length(tp_levels) - 1)
names(p_vals) <- tp_levels[-1]
for (i in 2:length(tp_levels)) {
this_tp <- tp_levels[i]
res <- wilcox.test(
baseline, mat[, this_tp],
paired = TRUE, exact = TRUE
)
p_vals[i-1] <- res$p.value
cat("T0 vs", this_tp, ": p =", signif(res$p.value, 4), "\n")
}
## Convert p-values to significance stars
p_stars <- ifelse(
p_vals < 0.001, "***",
ifelse(
p_vals < 0.01, "**",
ifelse(p_vals < 0.05, "*", "ns")
)
)
## Plot mean ± SD with base R
par(xaxs = "i", yaxs = "i")
bar_centers <- barplot(
mean_vals,
col    = bg_col,
```

```
border = NA,
ylim   = c(0, max(mean_vals + sd_vals) * 1.6),
xlab   = "",
ylab   = y_label,
main   = main_title,
cex.main = 1.6,
cex.lab  = 1.4,
axes   = FALSE,
xaxt   = "n"
)
axis(2, cex.axis = 1.2)
axis(1, at = bar_centers, labels = tp_levels, cex.axis = 1.3)
box()
lines(bar_centers, mean_vals, type = "b",
pch = 16, col = line_col, lwd = 2.5)
arrows(bar_centers, mean_vals - sd_vals,
bar_centers, mean_vals + sd_vals,
angle = 90, code = 3, length = 0.1,
lwd = 2.2, col = err_col)
## Stars only for T1–T4
target_bars <- bar_centers[2:length(tp_levels)]
star_y <- mean_vals[2:length(tp_levels)] +
sd_vals[2:length(tp_levels)] +
max(sd_vals, na.rm = TRUE) * 0.2
for (i in seq_along(p_stars)) {
if (p_stars[i] != "ns") {
text(target_bars[i], star_y[i], p_stars[i], cex = 1.6)
}
}
}
## Example calls for other biomarkers
plot_biomarker(
df,
var_name   = "LDH_U_L",
y_label    = "LDH (U/L)",
main_title = "Lactate Dehydrogenase Post-Match Response"
```

```
)

plot_biomarker(
df,
var_name   = "Myoglobin_ng_mL",
y_label    = "Myoglobin (ng/mL)",
main_title = "Myoglobin Post-Match Response"
)
plot_biomarker(
df,
var_name   = "IL6_pg_mL",
y_label    = "IL-6 (pg/mL)",
main_title = "Interleukin-6 Post-Match Response"
)
plot_biomarker(
df,
var_name   = "TNF_alpha_pg_mL",
y_label    = "TNF-α (pg/mL)",
main_title = "TNF-α Post-Match Response"
)
plot_biomarker(
df,
var_name   = "hsCRP_mg_L",
y_label    = "hs-CRP (mg/L)",
main_title = "High-sensitivity CRP Post-Match Response"
)
############################################################
## 4. Box-and-whisker + jitter plots (Figure 2 style)
############################################################
## If ggplot2 is not yet loaded:
## install.packages("ggplot2")

df <- read.csv("elitesoccer.csv", header = TRUE)

time_levels <- c("T0", "T1", "T2", "T3", "T4")
df$Timepoint <- factor(df$Timepoint, levels = time_levels)
```

```
markers <- c(
"CK_U_L",
"LDH_U_L",
"Myoglobin_ng_mL",
"IL6_pg_mL",
"TNF_alpha_pg_mL",
"hsCRP_mg_L"
)
long_list <- list()
marker_labels <- c(
CK_U_L          = "CK (U/L)",
LDH_U_L         = "LDH (U/L)",
Myoglobin_ng_mL = "Myoglobin (ng/mL)",
IL6_pg_mL       = "IL-6 (pg/mL)",
TNF_alpha_pg_mL = "TNF-α (pg/mL)",
hsCRP_mg_L      = "hs-CRP (mg/L)"
)
for (m in markers) {
tmp <- data.frame(
Subject_ID = df$Subject_ID,
Timepoint  = df$Timepoint,
Marker_raw = m,
Marker     = unname(marker_labels[m]),
Value      = df[[m]]
)
long_list[[m]] <- tmp
}

plot_data <- do.call(rbind, long_list)
## Avoid log10(0): add a small constant
plot_data$Value_adj <- plot_data$Value + 0.01
## Filter out invalid values before log10
plot_data_clean <- subset(
plot_data,
!is.na(Value_adj) & is.finite(Value_adj) & Value_adj > 0
)
```

```
p_box_jitter <- ggplot(
plot_data_clean,
aes(x = Timepoint, y = Value_adj)
) +
geom_boxplot(
width = 0.3,
outlier.shape = NA,
fill   = "grey85",
colour = "black"
) +
geom_jitter(
width = 0.12,
alpha = 0.5,
size  = 1
) +
scale_y_log10() +
facet_wrap(
~ Marker,
nrow   = 2,
scales = "free_y"
) +
labs(
x     = "Timepoint",
y     = "Concentration (log10 scale)",
title = "Distribution and skewness of acute biochemical responses\nacross five time points following match-play"
) +
theme_bw(base_size = 12) +
theme(
panel.grid       = element_blank(),
strip.background = element_rect(fill = "grey90", colour = NA),
strip.text       = element_text(face = "bold"),
plot.title       = element_text(hjust = 0.5, face = "bold"),
axis.title.x     = element_text(face = "bold"),
axis.title.y     = element_text(face = "bold")
)
print(p_box_jitter)
```

```
##########################################################
## 5. Peak %Δ heatmap for biochemical markers (Figure 3)
##########################################################
dat <- read.csv("elitesoccer.csv")
markers <- c(
"CK_U_L",
"LDH_U_L",
"Myoglobin_ng_mL",
"IL6_pg_mL",
"TNF_alpha_pg_mL",
"hsCRP_mg_L"
)
n_subj <- nrow(dat)
n_mark <- length(markers)
long_dat <- data.frame(
Subject_ID = rep(dat$Subject_ID, times = n_mark),
Timepoint  = rep(dat$Timepoint,  times = n_mark),
Marker     = rep(markers, each = n_subj),
Value      = as.vector(as.matrix(dat[, markers]))
)
baseline <- subset(long_dat, Timepoint == "T0")
baseline <- baseline[, c("Subject_ID", "Marker", "Value")]
names(baseline)[3] <- "Baseline"
long_pct <- merge(
long_dat, baseline,
by    = c("Subject_ID", "Marker"),
all.x = TRUE
)
long_pct$pct_change <- (long_pct$Value - long_pct$Baseline) /
long_pct$Baseline * 100
tmp_peak <- subset(long_pct, Timepoint != "T0")
peak_pct <- aggregate(
tmp_peak$pct_change,
by   = list(Subject_ID = tmp_peak$Subject_ID,
Marker     = tmp_peak$Marker),
FUN  = function(x) max(x, na.rm = TRUE)
```

```
)
names(peak_pct)[3] <- "peak_pct"
heat_df_peak <- aggregate(
peak_pct$peak_pct,
by   = list(Marker = peak_pct$Marker),
FUN  = function(x) mean(x, na.rm = TRUE)
)
names(heat_df_peak)[2] <- "mean_peak_pct"
heat_df_peak$Timepoint <- "Peak"
heat_df_peak$Marker <- factor(
heat_df_peak$Marker,
levels = markers,
labels = c("CK", "LDH", "Myoglobin", "IL-6", "TNF-α", "hs-CRP")
)
heat_df_peak$Timepoint <- factor(heat_df_peak$Timepoint, levels = "Peak")
max_val <- ceiling(max(heat_df_peak$mean_peak_pct, na.rm = TRUE) / 100) * 100
p_peak <- ggplot(heat_df_peak,
aes(x = Timepoint,
y = Marker,
fill = mean_peak_pct)) +
geom_tile(color = "white", linewidth = 0.5) +
scale_fill_gradientn(
colours = c("#F5F5F5", "#FDBB84", "#E34A33"),
limits  = c(0, max_val),
name    = "% change vs baseline"
) +
labs(
x     = NULL,
y     = NULL,
title = "Magnitude of peak biochemical responses following match-play\nexpressed as percent change from baseline"
) +
theme_minimal(base_size = 12) +
theme(
panel.grid      = element_blank(),
axis.text.x     = element_text(angle = 0, hjust = 0.5),
axis.text.y     = element_text(size = 11, face = "bold"),
```

```
plot.title      = element_text(hjust = 0.5, face = "bold"),
legend.position = "right"
)
print(p_peak)
###########################################################
## 6. Supplementary Figures S1–S7
###########################################################
## Figure S1: Individual trajectories (spaghetti plots) for Domain A markers
df_long_A <- df %>%
select(
Subject_ID, Timepoint,
CK_U_L, LDH_U_L, Myoglobin_ng_mL,
IL6_pg_mL, TNF_alpha_pg_mL, hsCRP_mg_L
) %>%
pivot_longer(
cols      = -c(Subject_ID, Timepoint),
names_to  = "Marker",
values_to = "Value"
)
df_long_A$Marker <- recode(
df_long_A$Marker,
"CK_U_L"          = "CK",
"LDH_U_L"         = "LDH",
"Myoglobin_ng_mL" = "Myoglobin",
"IL6_pg_mL"       = "IL-6",
"TNF_alpha_pg_mL" = "TNF-α",
"hsCRP_mg_L"      = "hs-CRP"
)
df_long_A$Marker <- factor(
df_long_A$Marker,
levels = c("CK", "LDH", "Myoglobin", "IL-6", "TNF-α", "hs-CRP")
)
p_s1 <- ggplot(df_long_A,
aes(x = Timepoint, y = Value, group = Subject_ID)) +
geom_line(color = "gray40", alpha = 0.5) +
facet_wrap(~ Marker, ncol = 3, scales = "free_y") +
```

```
theme_bw() +
labs(x = "Timepoint", y = NULL, title = NULL)
print(p_s1)
## Figure S2: Raincloud-style plots (violin + boxplot + jitter) for Domain A + B
df_long_AB <- df %>%
select(
Subject_ID, Timepoint,
CK_U_L, LDH_U_L, Myoglobin_ng_mL,
IL6_pg_mL, TNF_alpha_pg_mL, hsCRP_mg_L,
WBC_10e9_L, Neutrophil_pct, Lymphocyte_pct,
Lactate_mmol_L, Urea_mmol_L,
Creatinine_umol_L, Irisin_ng_ML = Irisin_ng_mL
) %>%
pivot_longer(
cols      = -c(Subject_ID, Timepoint),
names_to  = "Marker",
values_to = "Value"
)
df_long_AB$Marker <- recode(
df_long_AB$Marker,
"CK_U_L"          = "CK",
"LDH_U_L"         = "LDH",
"Myoglobin_ng_mL" = "Myoglobin",
"IL6_pg_mL"       = "IL-6",
"TNF_alpha_pg_mL" = "TNF-α",
"hsCRP_mg_L"      = "hs-CRP",
"WBC_10e9_L"      = "WBC",
"Neutrophil_pct"  = "Neutrophil%",
"Lymphocyte_pct"  = "Lymphocyte%",
"Lactate_mmol_L"  = "Lactate",
"Urea_mmol_L"     = "Urea",
"Creatinine_umol_L" = "Creatinine",
"Irisin_ng_ML"    = "Irisin"
)

df_long_AB$Marker <- factor(
```

```
df_long_AB$Marker,
levels = c(
"CK", "LDH", "Myoglobin", "IL-6", "TNF-α", "hs-CRP",
"WBC", "Neutrophil%", "Lymphocyte%", "Lactate",
"Urea", "Creatinine", "Irisin"
)
)
p_s2 <- ggplot(df_long_AB, aes(x = Timepoint, y = Value)) +
geom_violin(
fill   = "gray70",
color  = NA,
alpha  = 0.6,
trim   = FALSE,
width  = 0.8
) +
geom_boxplot(
width         = 0.2,
color         = "black",
fill          = NA,
outlier.shape = NA
) +
geom_jitter(
color  = "gray20",
size   = 1.2,
alpha  = 0.5,
width  = 0.15,
height = 0
) +
facet_wrap(~ Marker, scales = "free_y", ncol = 4) +
theme_bw() +
labs(x = "Timepoint", y = NULL, title = NULL)

print(p_s2)

## Figure S3A: Peak %Δ heatmap (Domain A) based on mat_S3A
## (Assumes mat_S3A has subjects in rows and markers in columns)
```

```
df_S3A <- melt(mat_S3A)
colnames(df_S3A) <- c("Subject_ID", "Marker", "DeltaPct")

p_S3A <- ggplot(df_S3A,
aes(x = Marker, y = Subject_ID, fill = DeltaPct)) +
geom_tile(color = "white") +
scale_fill_gradient2(
low  = "blue",
mid  = "white",
high = "red",
midpoint = 0,
name     = "%Δ"
) +
labs(
title = "Peak Δ% from Baseline (Domain A)",
x     = "",
y     = "Subject"
) +
theme_bw(base_size = 11) +
theme(
axis.text.x = element_text(angle = 45, hjust = 1),
panel.grid  = element_blank()
)
print(p_S3A)
## Figure S3B: Z-score heatmap (Domain B) based on mat_S3B
df_S3B <- melt(mat_S3B)
colnames(df_S3B) <- c("Subject_ID", "Marker", "Zscore")
p_S3B <- ggplot(df_S3B,
aes(x = Marker, y = Subject_ID, fill = Zscore)) +
geom_tile(color = "white") +
scale_fill_gradient2(
low  = "blue",
mid  = "white",
high = "red",
midpoint = 0,
```

```
name     = "Z-score"
) +
labs(
title = "Peak Value Z-scores (Domain B)",
x     = "",
y     = "Subject"
) +
theme_bw(base_size = 11) +
theme(
axis.text.x = element_text(angle = 45, hjust = 1),
panel.grid  = element_blank()
)
print(p_S3B)
############################################################
## Figure S5: Extended biomarker–performance scatter plots
## (Based on corr_df; assumes corr_df already created)
############################################################
marker_vars <- c(
"CK_U_L_delta",
"Myoglobin_ng_mL_delta",
"IL6_pg_mL_delta",
"hsCRP_mg_L_delta"
)
perf_vars <- c(
"WorstDelta_CMJ",
"WorstDelta_Sprint",
"WorstDelta_MVC",
"Peak_DOMS"
)
df_marker <- corr_df %>%
select(Subject_ID, all_of(marker_vars)) %>%
pivot_longer(
cols      = -Subject_ID,
names_to  = "Marker",
values_to = "MarkerChange"
)
```

```
df_performance <- corr_df %>%
select(Subject_ID, all_of(perf_vars)) %>%
pivot_longer(
cols      = -Subject_ID,
names_to  = "Performance",
values_to = "PerformanceChange"
)
df_scatter <- inner_join(df_marker, df_performance, by = "Subject_ID")
df_scatter$Marker <- factor(
df_scatter$Marker,
levels = marker_vars,
labels = c("ΔCK", "ΔMyoglobin", "ΔIL-6", "Δhs-CRP")
)
df_scatter$Performance <- factor(
df_scatter$Performance,
levels = perf_vars,
labels = c("Worst ΔCMJ", "Worst ΔSprint", "Worst ΔMVC", "Peak DOMS")
)
corr_labels <- df_scatter %>%
group_by(Marker, Performance) %>%
summarise(
r = suppressWarnings(
cor(
MarkerChange, PerformanceChange,
method = "spearman",
use    = "complete.obs"
)
),
p = suppressWarnings(
cor.test(
MarkerChange, PerformanceChange,
method = "spearman"
)$p.value
),
.groups = "drop"
) %>%
```

```
mutate(
label = paste0(
"ρ = ", sprintf("%.2f", r),
", p = ",
ifelse(p < 0.001, "<0.001", sprintf("%.3f", p))
)
)
p_S5 <- ggplot(df_scatter,
aes(x = MarkerChange, y = PerformanceChange)) +
geom_point(size = 1.8, colour = "black") +
geom_smooth(
method   = "lm",
se       = TRUE,
colour   = "black",
linewidth = 0.5
) +
facet_grid(Performance ~ Marker, scales = "free") +
geom_label(
data = corr_labels,
aes(label = label),
x = -Inf,
y = Inf,
hjust = -0.15,
vjust = 1.2,
size  = 3,
fill  = "white",
label.size  = 0.1,
alpha       = 0.85,
inherit.aes = FALSE
) +
theme_bw(base_size = 11) +
labs(
x = "Biomarker change (%Δ or worst change)",
y = "Performance / DOMS change"
) +
theme(
```

```
panel.grid = element_blank(),
strip.text = element_text(face = "bold"),
plot.title = element_text(face = "bold", hjust = 0.5)
)
print(p_S5)
########################################################
## Figure S6: Full time-course panels (mean ± SD) for all markers
########################################################
vars_to_plot <- c(
"CK_U_L","LDH_U_L","Myoglobin_ng_mL","IL6_pg_mL","TNF_alpha_pg_mL","hsCRP_mg_L",
"WBC_10e9_L","Neutrophil_pct","Lymphocyte_pct",
"Lactate_mmol_L","Urea_mmol_L","Creatinine_umol_L","Irisin_ng_mL",
"CMJ_cm","Sprint20m_s","MVC_N","RPE","DOMS"
)
df_summary <- df %>%
select(Timepoint, all_of(vars_to_plot)) %>%
pivot_longer(
cols      = -Timepoint,
names_to  = "Variable",
values_to = "Value"
) %>%
group_by(Variable, Timepoint) %>%
summarise(
mean = mean(Value, na.rm = TRUE),
sd   = sd(Value, na.rm = TRUE),
.groups = "drop"
)
df_summary$Variable <- recode(
df_summary$Variable,
"CK_U_L"            = "CK",
"LDH_U_L"           = "LDH",
"Myoglobin_ng_mL"   = "Myoglobin",
"IL6_pg_mL"         = "IL-6",
"TNF_alpha_pg_mL"   = "TNF-α",
"hsCRP_mg_L"        = "hs-CRP",
"WBC_10e9_L"        = "WBC",
```

```
"Neutrophil_pct"    = "Neutrophil%",
"Lymphocyte_pct"    = "Lymphocyte%",
"Lactate_mmol_L"    = "Lactate",
"Urea_mmol_L"       = "Urea",
"Creatinine_umol_L" = "Creatinine",
"Irisin_ng_mL"      = "Irisin",
"CMJ_cm"            = "CMJ",
"Sprint20m_s"       = "Sprint",
"MVC_N"             = "MVC",
"RPE"               = "RPE",
"DOMS"              = "DOMS"
)
df_summary$Variable <- factor(
df_summary$Variable,
levels = c(
"CK","LDH","Myoglobin","IL-6","TNF-α","hs-CRP",
"WBC","Neutrophil%","Lymphocyte%","Lactate","Urea","Creatinine","Irisin",
"CMJ","Sprint","MVC","RPE","DOMS"
)
)
p_s6 <- ggplot(df_summary,
aes(x = Timepoint, y = mean, group = 1)) +
geom_line(color = "black") +
geom_ribbon(
aes(ymin = mean - sd, ymax = mean + sd),
fill  = "gray70",
alpha = 0.4
) +
facet_wrap(~ Variable, scales = "free_y", ncol = 4) +
theme_bw() +
labs(x = "Timepoint", y = "Mean ± SD")

print(p_s6)

############################################################
## Figure S7: Multi-dimensional fatigue fingerprints (z-score heatmap)
```

```
## (Based on corr_df; assumes corr_df already created)
###########################################################

zfun <- function(x) {
if (all(is.na(x))) return(rep(NA, length(x)))
s <- sd(x, na.rm = TRUE)
m <- mean(x, na.rm = TRUE)
if (is.na(s) || s == 0) return(rep(0, length(x)))
(x - m) / s
}

fatigue_df <- corr_df %>%
select(
Subject_ID,
CK_U_L_delta,
IL6_pg_mL_delta,
hsCRP_mg_L_delta,
Irisin_ng_mL_delta = Irisin_ng_mL_delta,
WorstDelta_CMJ,
WorstDelta_Sprint,
WorstDelta_MVC,
Peak_DOMS
) %>%
mutate(
WorstDelta_CMJ_pos = -WorstDelta_CMJ,
WorstDelta_MVC_pos = -WorstDelta_MVC
) %>%
select(
Subject_ID,
CK_U_L_delta,
IL6_pg_mL_delta,
hsCRP_mg_L_delta,
Irisin_ng_mL_delta,
WorstDelta_CMJ_pos,
WorstDelta_Sprint,
WorstDelta_MVC_pos,
```

```
Peak_DOMS
)

colnames(fatigue_df) <- c(
"Subject_ID",
"ΔCK",
"ΔIL6",
"ΔCRP",
"ΔIrisin",
"WorstΔCMJ",
"WorstΔSprint",
"WorstΔMVC",
"PeakDOMS"
)

fatigue_z <- fatigue_df %>%
mutate(across(-Subject_ID, zfun))

fatigue_long <- fatigue_z %>%
pivot_longer(
cols      = -Subject_ID,
names_to  = "Metric",
values_to = "Zscore"
)

fatigue_long$Metric <- factor(
fatigue_long$Metric,
levels = c(
"ΔCK","ΔIL6","ΔCRP","ΔIrisin",
"WorstΔCMJ","WorstΔSprint","WorstΔMVC","PeakDOMS"
)
)

p_S7 <- ggplot(fatigue_long,
aes(x = Metric, y = Subject_ID, fill = Zscore)) +
geom_tile(color = "white") +
```

```
scale_fill_gradient2(
low      = "deepskyblue3",
mid      = "white",
high     = "firebrick3",
midpoint = 0,
name     = "Z-score"
) +
labs(
x     = "",
y     = "Player",
title = "Fatigue fingerprints across biochemical and performance domains"
) +
theme_bw(base_size = 11) +
theme(
panel.grid  = element_blank(),
axis.text.x = element_text(angle = 45, hjust = 1),
strip.text  = element_text(face = "bold"),
plot.title  = element_text(face = "bold", hjust = 0.5)
)
print(p_S7)
```
